# Real-time PCR assays that detect genes for botulinum neurotoxin A–G subtypes

**DOI:** 10.3389/fmicb.2024.1382056

**Published:** 2024-05-30

**Authors:** Segaran P. Pillai, Karen K. Hill, Jason Gans, Theresa J. Smith

**Affiliations:** ^1^Office of the Commissioner, Food and Drug Administration, Department of Health and Human Services, Silver Spring, MD, United States; ^2^Los Alamos National Laboratory, Bioscience Division, Los Alamos, NM, United States; ^3^Pathogen and Microbiome Institute, Northern Arizona University, Flagstaff, AZ, United States

**Keywords:** *Clostridium botulinum*, real-time PCR assay, serotype A-G assay, highly sensitive and specific assay, clinical diagnostics application, food testing, surveillance and outbreak investigation

## Abstract

The role of Real-Time PCR assays for surveillance and rapid screening for pathogens is garnering more and more attention because of its versatility and ease of adoption. The goal of this study was to design, test, and evaluate Real-Time TaqMan PCR assays for the detection of botulinum neurotoxin (*bont*/A-G) genes from currently recognized BoNT subtypes. Assays were computationally designed and then laboratory tested for sensitivity and specificity using DNA preparations containing *bont* genes from 82 target toxin subtypes, including nine bivalent toxin types; 31 strains representing other clostridial species; and an extensive panel that consisted of DNA from a diverse set of prokaryotic (bacterial) and eukaryotic (fungal, protozoan, plant, and animal) species. In addition to laboratory testing, the assays were computationally evaluated using *in silico* analysis for their ability to detect *bont* gene sequences from recently identified toxin subtypes. Seventeen specific assays (two for each of the *bont*/C, *bont*/D, *bont*/E, and *bont*/G subtypes and three for each of the *bont*/A, *bont*/B, and *bont*/F subtypes) were designed and evaluated for their ability to detect *bont* genes encoding multiple subtypes from all seven serotypes. These assays could provide an additional tool for the detection of botulinum neurotoxins in clinical, environmental and food samples that can complement other existing methods used in clinical diagnostics, regulatory, public health, and research laboratories.

## Introduction

Historically, methods employed for laboratory diagnostics, surveillance and detection of bacterial, viral, parasitic, and fungal infections, agents, and toxins were accomplished by culture and isolation of the causative organism followed by microscopy, cell-based assays, biochemical, antigen-based detection, serological-based analysis, or animal bioassays (American Society for Microbiology, 1985). In recent years, methods such as Polymerase Chain Reaction (PCR) have changed the paradigm of laboratory diagnostics, surveillance, and detection, and provided a tool for rapid diagnostics, and specific detection and surveillance of organisms via their genomic material. For example, PCR proved to be a powerful tool for detection, diagnosis, and epidemiological surveillance during the COVID-19 pandemic (Fernandes et al., 2020; Hossain et al., 2022).

Polymerase Chain Reaction-based assays for rapid detection have been further enhanced by the development of Real-Time PCR (RT-PCR), in which a dye-labeled “probe,” or short sequence of nucleotides, has been incorporated into the reaction with the forward and reverse primer. The dye-labeled probe then anneals to an internal homologous region within the PCR amplicon to provide greater specificity. RT-PCR also has the advantage that, instead of waiting until a complete PCR run has finished and the PCR products have been visualized on agarose gels, the results can be viewed in real time. If desired, the probe can be designed to target specific nucleotide(s), so that discriminating information between closely related gene sequences can be identified. The accretion of dye-labeled gene sequences at each PCR cycle provides rapid, real-time observation of results. Because PCR and RT-PCR assays are relatively inexpensive, rapid and reliable, these assays have been developed and adopted by the public health community for epidemiological surveillance and diagnostics of many infectious agents, such as *Bacillus anthracis*, *Francisella tularensis*, *Brucella* species, *Rickettsia*, *Coxiella burnetii*, *Yersinia pestis*, *Bordetella pertussis, Mycoplasma pneumoniae, Burkholderia pseudomallei*, monkeypox, Venezuelan equine encephalitis (VEE) virus, MERS-CoV, Ebola, and Zika.

Specific application of PCR or RT-PCR for the detection of genes associated with the production of botulinum neurotoxins (BoNTs) in clinical or environmental samples has proven to be valuable. Early PCR assays were developed as a rapid alternative to the animal-intensive mouse bioassay (Szabo et al., 1993; Franciosa et al., 1994; Hielm et al., 1996; Takeshi et al., 1996; Braconnier et al., 2001; Lindstrom et al., 2001). Later RT-PCR assays were designed and tested with both BoNT-producing and non-toxin producing clostridia for use in detecting botulinum neurotoxin genes in food and clinical samples, and for environmental sampling (Akbulut et al., 2004; De Medici et al., 2009; Fach et al., 2009; Hill et al., 2010; Kirchner et al., 2010). Currently, these assays are commonly used in animal botulism outbreak investigations and environmental screening related to prevention of animal botulism cases (Souillard et al., 2014, 2017; Skarin et al., 2015; Masters and Palmer, 2021; Nguyen et al., 2022; Park et al., 2022). They are also useful when screening environmental samples as potential sources for both infant and foodborne botulism (Shin et al., 2007; Sachdeva et al., 2010; Grenda et al., 2018; Maikanov et al., 2019), and today several standardized PCR assays (Anon, 2013, 2021) are being used in diagnostic and research laboratories for detection of *bont* genes in clinical and environmental samples, and contaminated foods. RT-PCR assays provide rapid presumptive evidence of botulinum neurotoxins through detection of *bont* genes, and interface with results of assays that detect the botulinum neurotoxin proteins, such as immunological assays, animal bioassays, or *in vitro* enzymatic activity assays, to confirm the presence of botulinum neurotoxins in a variety of clinical samples, foods, and environmental samples.

Botulinum neurotoxins are a diverse group of potent protein toxins that are produced by Gram positive anerobic spore-forming bacteria. The flaccid paralysis that results from botulism intoxication can have serious consequences if clinical intervention is not initiated in a timely manner. The seven serologically distinct botulinum neurotoxins, designated BoNT/A-G, are produced by seven distinct clostridial species: *C. parabotulinum, C. sporogenes, C. botulinum, C. novyi sensu lato, C. baratii, C. butyricum,* and *C. argentinense* (Smith et al., 2018) with some of the same toxin serotypes or subtypes being expressed in multiple clostridial species (Smith et al., 2023). Historically the BoNT-producing clostridia were taxonomically designated in different ways, such as *Bacillus botulinus*, *Clostridium botulinum*, and *Clostridium parabotulinum* (Smith et al., 2023). In 1953, it was proposed that any bacteria that produced botulinum neurotoxins should be called “*Clostridium botulinum*” (Prevot, 1953). *Clostridium botulinum* was then separated into “Group” designations to define the very different attributes of the strains within this “species” (Smith and Holdeman, 1968). Table 1 lists these Group designations with the accurate taxonomic nomenclatures for the neurotoxin-producing clostridia and the toxin types and subtypes they produce. Although the single species designation was created to prevent public health confusion, it concealed the true scientific taxonomic classification of the botulinum neurotoxin-producing clostridia; instead of a single species with various subspecies, there are seven distinct BoNT-producing species (Smith et al., 2018). Genomic sequencing and phylogenetic analysis of multiple strains has verified the status of the seven different genospecies (Suen et al., 1988a,b; Skarin et al., 2011; Weigand et al., 2015; Williamson et al., 2016). Interestingly, sequence analysis has identified the locations of botulinum neurotoxin genes within mobile genetic elements, indicating that the toxin genes are transferred into different bacteria by horizontal gene transfer (Williamson et al., 2016; Smith et al., 2020, 2021a,b).

**Table 1:** The seven BoNT-producing *Clostridium* species with their Group designations.

Genospecies	Group	Toxins produced
*C. parabotulinum*	I	A1–A8, B1–B3, B5–B8, F1–F5, F8–F9bivalents: A6B1, A2B3, A2B5, A2B7, A2F4, A2F5, A2F4F5, B5A4, and B5F2chimera: HA
		*C. sporogenes*	I	B1, B2, B5, B6
		*C. botulinum*	II	B4, E1–E12, F6
*C. novyi sensu lato*	III	C, D chimeras: CD, DC
*C. argentinense*	IV	G
*C. butyricum*		E4, E5
*C. baratii*		F7

The relationship of the Group and genospecies designations, and the botulinum neurotoxin types expressed by each are listed.

The sequencing of *bont* genes has led to the discovery of significant variation among and within the BoNT serotypes. Serotype-level differences among the *bont*/A-G genes range from 24 to 42% and the BoNT proteins encoded by these genes differ by 36–68%. Table 2 illustrates the range of diversity among the toxin genes and proteins. The level of nucleotide variation observed among the serotypes requires the development of individual serotype-specific PCR assays, and illustrates the challenges associated with designing assays for the detection of *bont* genes encompassing all known toxin serotypes, subtypes, and subtype variants.

**Table 2:** The percent identities of nucleotides and amino acid residues among representative toxin subtypes from each BoNT serotype.

	Percent identity nucleotides								
Subtype	A1	B1	C	D	E3	F1	F5	F7	G
A1	—	62%	59%	60%	63%	64%	63%	64%	32%
B1	39%	—	60%	61%	61%	62%	62%	63%	**72%**
C	32%	33%	—	**70%**	59%	59%	58%	59%	60%
D	33%	35%	**52%**	—	60%	60%	60%	61%	60%
E3	40%	38%	33%	34%	—	**76%**	**72%**	**76%**	61%
F1	40%	39%	34%	34%	**64%**	—	**82%**	**82%**	61%
F5	39%	39%	33%	34%	**57%**	**70%**	—	**76%**	61%
F7	41%	41%	33%	34%	**64%**	**74%**	**74%**	—	61%
G	39%	**57%**	34%	35%	38%	38%	38%	39%	—
	Percent identity amino acid residues								

Identities were determined using the BLAST Global alignment tool for nucleotide sequences based on the Needleman-Wunsch algorithm (Needleman and Wunsch, 1970). Three BoNT/F representatives are included to demonstrate the relationship between the diverse subtypes in addition to relationships between certain serotypes. Percent identities with closely related serotypes (C,D, B–G, and E,F), and the BoNT/F subtypes are shown in bold text with gray highlighting.

Additional variation is observed within the serotypes, which are referred to as subtypes that are indicated with numeric designations (e.g., A2, B5, etc.). The toxin subtypes are generally defined as having a minimum of 2.6% difference in amino acid residues (Peck et al., 2017), which is a structural distinction based on nucleotide differences. Subtype nucleotide differences may be as small as 0.5%, as with *bont/*E1 and *bont/*E2, or quite large, such as the 23.7% difference between *bont/*F5 and *bont/*F7. A notable exception involves the genes encoding BoNT/G, which are highly conserved, and having no toxin subtypes. Additional variation is seen through the examination of chimeric toxins, such as CD or DC toxins, which are composed of two toxin subtypes or serotypes that have emerged through various recombination events. The underlying genetic differences among the subtypes are illustrated in Table 3.

**Table 3:** Nucleotide variations among genes encoding BoNT/A **(A)**, /B **(B)**, /C and /D **(C,D)**, /E **(E)**, and /F **(F)** subtypes.

(A)	Nucleotide differences between subtypes (%)							
	A1	A2	A3	A4	A5	A6	A7	A8
A1	—	5.4	7.9	5.7	**1.4**	2.2	3.2	3.4
A2		—	3.5	6.3	5.1	4.5	5.5	4.0
A3			—	**8.2**	7.6	7.0	8.0	6.7
A4				—	6.2	6.2	6.8	5.7
A5					—	2.2	2.9	3.3
A6						—	3.6	3.6
A7							—	4.4
A8								—

(B)	Nucleotide differences between subtypes (%)							
	B1	B2	B3	B4	B5	B6	B7	B8
B1	—	2.4	2.0	3.7	1.9	2.1	2.4	2.3
B2		—	0.9	3.7	2.7	**0.8**	2.4	2.1
B3			—	3.6	2.3	1.1	2.2	2.0
B4				—	3.9	**4.0**	3.7	3.9
B5					—	2.6	2.8	2.7
B6						—	2.7	1.9
B7							—	2.7
B8								—

(C,D)	Nucleotide differences between subtypes (%)			
	C1	CD	D	DC
C	—	6.3	**17.5**	13.6
CD		—	6.9	15.9
D			—	**3.4**
DC				—

(E)	Nucleotide differences between subtypes (%)											
	E1	E2	E3	E4	E5	E6	E7	E8	E9	E10	E11	E12
E1	—	**0.5**	0.7	1.5	1.7	1.5	0.9	2.1	5.8	2.3	3.2	3.9
E2		—	1.3	1.7	2.0	1.7	1.5	1.5	5.8	2.2	2.9	3.8
E3			—	2.1	2.4	2.0	1.2	2.4	6.0	2.7	3.6	4.1
E4				—	2.7	1.7	1.9	2.0	5.2	2.5	3.3	3.8
E5					—	2.7	2.6	3.0	5.5	3.2	3.9	3.5
E6						—	1.8	5.8	**6.1**	2.2	3.2	4.8
E7							—	1.1	5.8	1.6	3.1	4.1
E8								—	5.8	1.0	2.6	4.4
E9									—	5.7	5.5	4.7
E10										—	2.1	4.3
E11											—	4.6
E12												—

(F)	Nucleotide differences between subtypes (%)								
	F1	F2	F3	F4	F5	F6	F7	F8	F9
F1	—	8.7	8.1	4.0	18.8	6.4	17.8	2.3	8.0
F2		—	**1.1**	7.8	15.2	4.5	19.8	8.3	3.8
F3			—	7.7	15.2	4.6	20.2	8.1	3.8
F4				—	17.8	6.3	18.6	3.6	7.7
F5					—	15.7	**23.7**	18.1	15.6
F6						—	19.5	6.4	5.9
F7							—	18.0	19.7
F8								—	8.0
F9									—

Percent identities were generated with a representative sequence for each subtype and determined using the BLAST Global alignment tool for nucleotide sequences based on the Needleman-Wunsch algorithm (Needleman and Wunsch, 1970). Values in bold text with gray highlighting indicate the range of variation within *bont*/A (1.4–8.2%), *bont*/B (0.8–4.0%), *bont*/C and /D (3.4–17.5%), *bont*/E (0.5–6.1%), and *bont*/F (1.1–23.7%).

The aim of this project was to develop assays specific for each of the seven toxin serotypes and evaluate these assays for their ability to detect genes from all currently identified BoNT subtypes within each serotype. The assays were designed using a comprehensive analysis of the available *bont* gene sequences resident in the public GenBank database as nucleotide sequences or as part of plasmid or genomic sequence data. The derived assay designs were experimentally assessed for sensitivity and specificity and then re-evaluated by *in silico* computational analysis. The intended application of these nucleic acid-based RT-PCR assays is to rapidly detect botulinum neurotoxin genes and supplement existing protein-based toxin assays such as ELISA, mouse bioassay, and Endo-PEP mass spectrometry. Together, these assays will provide complementary results confirming the presence of botulinum neurotoxins. The details of the design, laboratory testing, and *in silico* evaluation of assays to detect *bont/*A-G genes are presented below.

## Materials and methods

### *In silico* assay designs

Assay development included the design of PCR primers and VIC or FAM dye-labeled, minor groove binder (MGB) probes that are specific to each toxin serotype and the cycling conditions that were used to test the assays. MGB probes have a covalently attached chemical group that increases the perfect-match binding affinity to complementary DNA, enabling the design of probes that are shorter than traditional TaqMan probes (Kutyavin et al., 2000). Shorter probes are useful when attempting to find a perfectly conserved sequence region in a set of diverse target sequences. The primers and probes were computationally designed using unpublished assay design software developed at Los Alamos National Laboratory. The computational design process utilized 150 available botulinum neurotoxin gene sequences representing *bont*/A-G. Candidate assays predicted to detect the target *bont* sequences were then queried against all publicly available nucleic acid sequences (in 2011), including the GenBank “nt” database (containing viral, prokaryotic, and eukaryotic nucleotide sequences) and the GenBank whole genome shotgun (WGS) bacterial genome assemblies to determine specificity and identify nonspecific positive reactions. Assays that passed initial *in silico* specificity screening were then experimentally tested in the laboratory.

Three assays that were designed for each set of *bont*/A, *bont/B*, and *bont/F* subtype genes and two assays that were designed for each set of *bont*/C, *bont*/D, *bont*/E, and *bont*/G genes were subjected to further testing and analysis. The three different assays that were designed for *bont*/F were targeted to identify distinct *bont*/F subtypes due to the diversity (1.1–23.7%) that is observed among them (Table 3). The assays that were developed for *bont*/B included an assay that was designed to discriminate between *bont*/B and the silent unexpressed *bont*/(B) gene that contains a premature stop codon due to a mutation (Hutson et al., 1996); this assay design has forward and reverse primers and two probes in the reaction for allelic discrimination. The assay design for Cb(B)stop targets the nucleotide mutation responsible for the stop codon in the silent B sequences. Cb(B)stop starts at bp 342 and stops at bp 416, with the locations specified relative to the *bont*/B gene in BoNT/B1 okra, GenBank accession CP000940. The SNP bases that discriminate between active and silent B strains are shown in lower case/bold/underlined in Table 4. The two probes in the reaction have either the FAM dye that provides the detection of the *bont/*(B) or the VIC dye that detects genes encoding full-length toxins from various *bont/*B subtypes.

**Table 4:** *bont/*A-G assay designs.

		Primer and Probe Sequence (5′-3′)	Amplicon Location (start-stop)	Sequence in strain:
***bont*/A Assays**	**CbA 2.341**	F: ATCTTACGCGAAATGGTTATGG**Y**TCTAC R: AGGATTTGTATCAACTTCAAGTGACTCC P: CAATACATTAGATTTAGCCC-MGB	521–618	BoNT/A1 Hall (CP000727)	**CbA 4.16566**	F: GATCCAGC**R**GTA**R**CATTAGCACATGA R: AAGTTCCTCAA**RR**CTTACTTCTAACCCA P: ACCCTATTTG**K**ATTAATTGCT-MGB	646–789	**CbA 2.323**	F: ATCTTACGCGAAATGGTTATGG**Y**TCTAC R: AAGTTCATGTGCTAATG**Y**TAC**Y**GCTGGA P: TTGAAGTTGATACAAATCCT-MGB	521–675
	***bont*/B Assays**	**CbB 2.11**	F: ACCTTATCTTGGAGATAGACGTGTTCCA R: AAACTGGCCCAGGTCCAAATATT P: CAAACATTGCTAGTGTAACTG-MGB		348–499	BoNT/B1 okra (CP000940)	**CbB 2.295**	F: CGTGTTCCACTCGAA**K**AGTTTAACACA R: T**Y**GCTCCACTTCTCCTGGATTACT P: ACATTGCTAGTGTAACTGTTA-MGB	367–450	**Cb(B) stop**	F: TGGTATACCTTATCTTGGAGATAGACG R: TTAACAGTTACACTAGCAATGTTTGTG P(silent/FAM): TCCACTCGAA**t**AGTTTAA-MGB P(active/VIC): TCCACTCGAA**g**AGTTTAA-MGB	342–416
		***bont*/C Assays**	**CbC 2.11**		F: AATGTGGGCAAATGATGTAGTTGAAGA R: CACC**R**GTAACTGCAAATGCTTCAGTAAA P: AGATGTATCAGCTATTATTCC-MGB		1,797–1,966	BoNT/C1 Stockholm (CP063817)	**CbC 2.968**	F: TTTACGA**S**ATCAATTGAGGA**R**GCTTTGG R: CCACCTTGAACACCCG**Y**ATTTACTTT P: AGCTAGTKTAGGAAAGTAAGT-MGB	1,690–1,787
***bont*/D Assays**			**CbD 2.0**	F: AAATCCATCATTTGAAGGGTTTGGAACA R: TGATGCAAAGAATGTGTTAACTCATGCA P: ACAGCTGAACTTTGATTAGAT-MGB	537–710		BoNT/D 1873 (CP063823)		**CbD 2.276**	F: GGAGATTCAAGTACGCCTGAAGATACAT R: AGTGGTCCAAATATCAATACACTTGGTG P: AATGGTAGTTGGAAAGTAACA-MGB	352–485
	***bont*/E Assays**	**CbE 2.0**	F: AATAGTGAATCAGCACCTGGACTTTCA R: TTCACCTTCGGGCACTTTCTGT P: TGATTCTAATGGAACAAGTGA-MGB	1,426–1,575	BoNT/E3 Alaska E43 (CP001078)	**CbE 2.693**		F: CACAGAAAGTGCCCGAAGGTG R: GCTGCTTGCACAGGTTTATTGACATTAT P: **M**TCTTCAATTGATACAGCATT-MGB	1,574–1,709
***bont*/F Assays**		**CbF 2.0**	F: AGATCCTGCAATTTCACTAGC**Y**CATGA R: CGGCTATCATAAGAGGTSCT**Y**GC P: CACT**R**CATGGATTATACGG-MGB	657–775		BoNT/F1 Langeland (CP000728)	**CbF 2.161**	F: AAGGATTTGGATCAATTCAGCTCATGTC R: AACACCTTTAGCTCCGTATAAACCATGT P: CATGAGCTAATGATATTGCAG-MGB	552–702	BoNT/F7 Sullivan (CP006905)	**CbF 2.1084**	F: AGGAGGCATTTGAATTATTAGGAGCG R: ATTTCTTTCCACTTTGCTTCTCTTTCG P: AATTACAGGRATTGYAAGCTC-MGB	1,913–2,090	BoNT/F5 CDC 54074 (GU213211)
	***bont*/G Assays**	**CbG 2.0**	F: AAACATTTGGGCTGTTTGGAATTGGTAA R: ATCCACGGGAATGAATTGCCAATTACAT P: CATAAGTCAGTGGTATCTCAG-MGB	3,713–3,876	BoNT/G 89G (CP014175)	**CbG 2.83**	F: ACGGAATTTAATGGTCAGAATAAGGCGG R: CATTACAGGCTTGCACATTGCTATTCTA P: AGAGGCTTATGAAGAAATCAG-MGB	1,216–1,320
		**Bacterial positive control**	**16S rRNA**	F: CCTACGGG**D**GGC**W**GCA R: GGACTAC**HV**GGGT**M**TCTAATC P: CAGCAGCCGCGGTA-MGB		2,730,352–2,730,817	*E. coli* K-12 (MG1655)

The primer and probe sequences, and sequence locations, for the *bont/A-G* assay designs are shown. All probes contain a Minor Groove Binding (MGB) moiety. Degenerate bases are in bold font and underlined (**Y** = C or T; **R** = A or G; **M** = A or C; **K** = T or G; **S** = G or C; **D** = A, T, or G; **W** = A or T; **H** = A, T, or C; **V** = A, G, or C). The SNP bases (t and g) that discriminate between the active and silent BoNT/B in the Cb(B) stop assay are underlined in bold lower-case font.

### Laboratory evaluation of the RT-PCR assays

The assay primers and probes listed in Table 4 were laboratory evaluated using DNA from 82 BoNT-producing strains, plus panels containing DNA from clostridia that lack *bont* genes, and additional prokaryotic and eukaryotic DNA preparations. DNA was purified using phenol/chloroform extraction (Hill et al., 2007) or the Qiagen DNeasy Blood and Tissue kit, and was quantified using spectrophotometry. All laboratory experiments were performed using an ABI 7500 Fast Instrument in 96-well format using the cycling conditions of 95°C for 20 s followed by 40 cycles of 95°C for 3 s, 60°C for 30 s. Ct values of less than 40 indicate detection. The PCR reagents included TaqMan Fast Universal PCR Master Mix with No AmpErase UNG, with 900 nM of forward and reverse primers and 250 nM of probe. All assay testing was performed in triplicate.

### Sensitivity testing using *bont*-containing *Clostridium* strains

The sensitivity/specificity panel consisted of DNA preparations from a total of 82 *bont*-containing *Clostridium* strains: 21 BoNT/A strains containing *bont*/A1-A4 genes, including BoNT/A1(B) strains and two bivalent strains (BoNT/A2B5 and BoNT/B5A4); 34 BoNT/B strains containing *bont*/B1-B5 genes, including five A1(B) strains and four bivalent strains (BoNT/A2B5, BoNT/B5A4, and 2 BoNT/B5F2); 10 BoNT/C strains containing both *bont*/C and *bont*/CD genes; three BoNT/D strains; nine BoNT/E strains containing *bont*/E1, /E3, and /E4 genes; six BoNT/F strains containing *bont*/F1, /F2, /F6, and /F7, including two bivalent strains (2 BoNT/B5F2); and six BoNT/G strains. Supplementary Table S1 lists the botulinum neurotoxin-producing strains used in both sensitivity and specificity testing. Sensitivity testing of the assays utilized 1 pg. of DNA (approximately 200–400 genome copies) from strains containing the target *bont* gene.

### Specificity testing using *bont-*containing *Clostridium* strains

Specificity testing of the assays was conducted with DNA preparations containing non-target *bont* genes using a total of 100 pg. of DNA per sample. The strains within the sensitivity/specificity panel were selected to encompass the available *bont* subtypes within a toxin serotype. To ensure assay performance with each primer-probe set, positive controls using target DNA were included. A 16S rRNA TaqMan assay (Liu et al., 2012) was included as an amplification control for each DNA sample.

### Specificity testing using non-neurotoxinogenic clostridial species

Additional specificity testing of the assays was performed with a panel of 31 bacterial strains representing 16 non-neurotoxigenic clostridial species to determine potential nonspecific reactions (Supplementary Table S2). Strain DNA preparations of these clostridial species were tested individually at 100 pg. concentrations. Serotype-specific positive controls were added for each assay run, and a 16S rRNA assay was used to verify DNA amplification for each individual sample preparation.

### Specificity testing using DNA from other bacterial species

Commercially available DNA preparations from 110 bacterial species were tested in pools containing 10 bacterial species/pool. Supplementary Table S3 lists the individual samples and their pools. Each bacterial species within the pool is represented by 100 pg. DNA per well. Positive controls containing serotype-specific *bont* genes were added for each assay run, and a 16S rRNA assay was used to verify DNA amplification for each individual sample preparation.

### Specificity testing using DNA from fungi/yeast and protozoan species

Commercially available DNA preparations from 12 fungi/yeast and protozoan samples were tested in pools of four samples/pool. Supplementary Table S4 lists the individual fungi/yeast and protozoa samples and their pools. Positive controls containing serotype-specific *bont* genes were added for each assay run, and 100 pg. of *E. coli* K12 DNA was added to each pool which was then tested using a 16S rRNA assay to verify DNA amplification.

### Specificity testing using DNA from additional eukaryotes

Commercially available DNA preparations from 25 samples of eukaryotes representing fish, birds, animals and plants (Supplementary Table S5) were tested in seven pools with each pool containing 1–4 species. Positive controls containing serotype-specific *bont* genes were added for each assay run, and 100 pg. of *E. coli* K12 DNA was added to each pool, which was then tested using a 16S rRNA assay to verify DNA amplification.

### *In silico* evaluation of TaqMan assays

Because of the difficulty in acquiring strains representing all subtypes, *in silico* assay evaluations were performed after laboratory screening to evaluate assay performance with newer identified toxin subtypes whose sequences were not available during the original computational design process in 2011.

Because representatives of all strain subtypes were not available, the ability of the assays to detect *bont*/A5-/A8; *bont*/B6-/B8; *bont*/DC; *bont*/E2 and E5-/E12; *bont*/F3-/F5 and *bont*/F8-/F9, as well as currently identified toxin subtype variants, were computationally evaluated using standard nucleotide BLAST analyses. Multiple BLAST analyses performed in 2022 through 2024 focused on the identification of positive target *bont* genes by specific assays. *In silico* assay screening was also performed to determine possible false positive reactions using the open source thermonucleotideBLAST (version 2.5) software (Gans and Wolinsky, 2008). All assay designs (defined by the primers and probes listed in Table 4) were searched against all complete and whole genome shotgun (WGS) clostridial genome sequences that were available in GenBank (as of November 2022). Matches required predicted primer-template melting temperatures ≥45°C and a predicted probe-template melting temperature ≥ 30°C. Note that the thermonucleotideBLAST software does *not* explicitly account for probe MGB moieties when computing melting temperatures (which are assumed to increase probe melting temperatures by 20°C). While the *in silico* screening did not identify any potential assay false negatives when the assays were originally designed in 2011, a number of mismatches between assay oligos and target sequences were computationally identified when the assays were rescreened in 2022, 2023, and 2024, and are documented in the results section below. The computational identification of potential assay failures (due to potential false positives or false negatives), often referred to as “assay erosion” (Sozhamannan et al., 2015), is a result of designing detection assays based on incomplete knowledge of microbial sequence diversity (i.e., the limited number of *bont*/A-G that were available in 2011). As new sequences are deposited in public sequence databases, assay failures due to both existing, but previously unknown, sequence variants, and recently evolved sequences can be detected by computational screening. It should be noted that *in silico* evaluations are predictive tools and, whenever possible, these predictions should be verified using laboratory testing with purified DNA preparations.

### *In silico* evaluation of ISO 17919:2013 assays

The ISO/TC 17919:2013 document describes several procedures that are intended to serve as International Standard methods for the detection of genes from BoNT/A, BoNT/B, BoNT/E, and BoNT/F in food, feed, and environmental samples (Fenicia et al., 2011). *In silico* evaluations of the PCR and RT-PCR assays contained in the ISO/TC 17919:2013 (Anon, 2021) were performed here for comparative purposes. The *in silico* analyses were done using both the BLAST and thermonucleotideBLAST methods listed above. A total of five assays (two *bont*/A assays and one each *bont*/B, *bont*/E, and *bont*/F) involving agarose gel electrophoresis (Lindstrom et al., 2001) and eight RT-PCR assays (two each *bont*/A *bont*/B, *bont*/E, and *bont*/F) (De Medici et al., 2009; Fach et al., 2011; Fenicia et al., 2011) that were developed between 2001 and 2009 were evaluated.

Two multiplex agarose gel assays were described in the ISO/TC 17919:2013 that utilized two different *bont*/A primer sets but identical *bont*/B, *bont*/E, and *bont*/F primer sets (Lindstrom et al., 2001; De Medici et al., 2009), and two RT-PCR methods are also described that utilize different primer/probe sets. One primer/probe set Fach Type (A–F) is referenced in Fach et al. (2009), but we were unable to determine the origin of the second set (CBOT A-F). Thus, we performed *in silico* analyses on a total of 13 different primer or primer/probe sets for these comparisons. The methods referenced in ISO/TC 17919:2013 were developed and published between 2001 and 2011.

## Results

### Results for *bont*/A assays

#### Assay design

With the exception of chimeric toxins, the *bont/A* genes are second only to *bont/*F genes in diversity, with up to 8.2% difference in nucleotides between subtypes (Table 3A). This genetic diversity presents challenges when designing PCR-based assays that can detect genes from all BoNT/A subtypes. The assay designs for *bont/*A-containing strains were developed using 31 DNA sequences from *bont* genes representing BoNT/A1-A6 subtypes, including BoNT/A1(B), and bivalent BoNT/A6B1, BoNT/A2B5, and BoNT/B5A4 subtypes. Three assay designs, based on primer/probe sets CbA 2.323, CbA 2.341, and CbA 4.16566 (detailed in Table 4, *bont*/A), were experimentally tested for sensitivity and specificity, and were subsequently computationally evaluated for potential binding to genes from additional BoNT/A1-A8 strains.

#### RT-PCR sensitivity testing

The three designed assays were experimentally tested in triplicate experiments with DNA preparations from 21 *bont*/A-containing strains that included DNA sequences from BoNT/A1-A4, BoNT/A1(B), and bivalent BoNT/A2B5 and BoNT/B5A4 subtypes. Sensitivity testing was done using 1 pg. of target DNA per assay. The results, summarized in Table 5 and detailed in Supplementary Table S6A, indicate that all three assays successfully detected all target *bont*/A-containing strains.

**Table 5:** Summary of laboratory sensitivity and specificity testing of *bont* gene RT-PCR assays.

BoNT subtype	CbA 2.323	CbA 4.16566	CbA 2.341	CbB 2.11	CbB 2.295	Cb(B) stop	CbC 2.11	CbC 2.968	CbD 2.0	CbD 2.276	CbE 2.0	CbE 2.693	CbF 2.0	CbF 2.161	CbF 2.1084	CbG 2.0	CbG 2.83
A1	**16**	**16**	**16**	0	0	0	0	0	0	0	0	0	0	0	0	0	0
A2	**3**	**3**	**3**	0	0	0	0	0	0	0	0	0	0	0	0	0	0
A3	**1**	**1**	**1**	0	0	0	0	0	0	0	0	0	0	0	0	0	0
A4	**1**	**1**	**1**	0	0	0	0	0	0	0	0	0	0	0	0	0	0
B1	0	0	0	**9**	**9**	**9-VIC**	0	0	0	0	0	0	0	0	0	0	0
B2	0	0	2*	**13**	**13**	**13-VIC**	0	0	0	0	0	0	0	0	0	0	0
B4	0	0	0	**3**	**3**	**3-VIC**	0	0	0	0	0	0	0	0	0	0	0
B5	0	0	0	**4**	**4**	**4-VIC**	0	0	0	0	0	0	0	0	0	0	0
(B)	0	0	0	**5**	**5**	**5-FAM**	0	0	0	0	0	0	0	0	0	0	0
C	0	0	6*	0	0	0	**9**	**9**	0	0	0	0	0	0	0	0	0
CD	0	0	1*	0	0	0	**2**	**2**	0	0	0	0	0	0	0	0	0
D	0	0	2*	0	0	0	0	0	**3**	**3**	0	0	0	0	0	0	0
E1	0	0	0	0	0	0	0	0	0	0	**4**	3*	0	0	0	0	0
E2	0	0	0	0	0	0	0	0	0	0	**2**	1*	0	0	0	0	0
E3	0	0	1*	0	0	0	0	0	0	0	**2**	**2**	0	0	0	0	0
E4	0	0	0	0	0	0	0	0	0	0	**1**	0*	0	0	0	0	0
F1	0	0	1*	0	0	0	0	0	0	0	0	0	**4**	0	0	0	0
F2	0	0	0	0	0	0	0	0	0	0	0	0	**2**	0	**2**	0	0
F6	0	0	0	0	1*	0	0	0	0	0	0	0	**1**	0	0	0	0
F7	0	0	0	0	0	0	0	0	0	0	0	0	0	**1**	0	0	0
G	0	0	0	0	0	0	0	0	0	0	0	0	0	0	0	1*	**6**

Values indicate the number of DNA preparations tested that were positive for each toxin subtype. The Cb(stop) assay discriminates between active and inactive (silent) bont/B genes using VIC (active) or FAM (silent) dyes. FAM dye results were tested with 1 pg. of DNA; VIC dye results were tested with 100 pg. of DNA. Results shown in black font are based on 1 pg. DNA (sensitivity testing); nonspecific results shown in red font were tested with 100 pg. DNA (specificity testing). The results are based on triplicate testing. ^*^Indicates results where triplicate test results were variable, including at least one negative result.

#### RT-PCR specificity testing

Specificity testing results showed that the *bont*/A assays CbA 2.323 and CbA 4.16566 did not amplify DNA from alternative *bont*-containing strains, other clostridial species, or additional prokaryotic and eukaryotic species when 100 pg. of DNA were used (Table 5). Results with assay CbA 2.341 indicated some non-specificity when tested against alternative *bont*-containing strains, with positive results with DNA from two BoNT/B2 strains, multiple BoNT/C and BoNT/D strains, one BoNT/E3 strain, and one BoNT/F1 strain, with negative and variable Ct values ranging between 36.2 and 39.2 (Table 5). Positive controls for the assays included BoNT/A1(B) Hall 3676 and BoNT/B5A4 strain 657.

#### *In silico* evaluation of additional BoNT/A subtypes

The assays were later evaluated *in silico* against genes from all available BoNT/A1-A8 subtypes, including bivalent BoNT/A6B1, BoNT/A2B5, BoNT/A2F4, BoNT/A2F5, and BoNT/B5A4 strains. The *in silico* analysis showed that all three assays were capable of detecting *bont* genes from additional BoNT/A1-A8 subtypes that had not been experimentally evaluated, with perfect matches against the forward primers, reverse primers, and probes for the available serotype A strains, with one exception (Table 6A). There was a single G→A mismatch in the reverse primer of the CbA 4.16566 assay with *bont*/A8 genes, predicting a possible assay failure.

**Table 6:** Summaries of *in silico* analysis results using the primer/probe sets in Table 4, listed by subtype.

		CbA 2.323		CbA 2.341		CbA 4.16566	
A		Predicted positive	Predicted negative	Predicted positive	Predicted negative	Predicted positive	Predicted negative
Subtype	# analyzed	(exact match)	(mismatches)	(exact match)	(mismatches)	(exact match)	(mismatches)
A1	125	125	0	125	0	125	0
A1(B)	61	61	0	61	0	61	0
A2	148	148	0	148	0	148	0
A3	12	12	0	12	0	12	0
A4	3	3	0	3	0	3	0
A5	6	6	0	6	0	6	0
A6	2	2	0	2	0	2	0
A7	1	1	0	1	0	1	0
A8	2	2	0	2	0	0	2
							
		CbB 2.11		CbB 2.295		Cb(B) stop	
B		Predicted positive	Predicted negative	Predicted positive	Predicted negative	Predicted positive	Predicted negative
Subtype	# analyzed	(exact match)	(mismatches)	(exact match)	(mismatches)	(exact match)	(mismatches)
A1(B)	62	62	0	62	0	62	0
B1	9	9	0	9	0	9	0
B2	91	87	4	87	4	87	4
B3	32	32	0	32	0	32	0
B4	73	73	0	73	0	73	0
B5	19	19	0	19	0	19	0
B6	4	4	0	4	0	4	0
B7	11	11	0	11	0	11	0
B8	3	3	0	0	3	3	0

		CbC 2.11		CbC 2.968	
C		Predicted positive	Predicted negative	Predicted positive	Predicted negative
Subtype	# analyzed	(exact match)	(mismatches)	(exact match)	(mismatches)
C	13	13	0	13	0
CD	54	54	0	54	0
CDDC	1	1	0	1	0
D	2	0	2	0	(2)
DC	48	0	48	0	(48)
					
		CbD 2.0		CbD 2.276	
D		Predicted positive	Predicted negative	Predicted positive	Predicted negative
Subtype	# analyzed	(exact match)	(mismatches)	(exact match)	(mismatches)
D	2	2	0	2	0
DC	48	48	0	48	0
C	13	0	13	0	(13)
CD	54	0	54	0	(54)
CDDC	1	0	1	0	(1)
		CbE 2.0		CbE 2.693	
E		Predicted positive	Predicted negative	Predicted positive	Predicted negative
Subtype	# analyzed	(exact match)	(mismatches)	(exact match)	(mismatches)
E1	79	79	0	79	0
E2	5	5	0	5	0
E3	106	106	0	106	0
E4	6	6	0	6	0
E5	15	15	0	15	0
E6	8	8	0	8	0
E7	2	2	0	2	0
E8	1	0	1	0	1
E9	1	1	0	0	1
E10	45	45	0	45	0
E11	9	9	0	0	9
E12	1	1	0	0	1

		CbF 2.0		CbF 2.161		CbF 2.1084	
F		Predicted positive	Predicted negative	Predicted positive	Predicted negative	Predicted positive	Predicted negative
Subtype	# analyzed	(exact match)	(mismatches)	(exact match)	(mismatches)	(exact match)	(mismatches)
F1	16	16	0	0	16	0	16
F2	14	14	0	0	14	14	0
F3	3	3	0	0	3	3	0
F4	19	19	0	0	19	0	19
F5	10	0	(10)	0	(10)	10	0
F6	18	18	0	0	18	0	0
F7	12	0	(12)	12	0	0	12
F8	1	1	0	0	1	0	1
F9	1	1	0	0	1	0	1
							
		CbG 2.0		CbG 2.83			
G		Predicted positive	Predicted negative	Predicted positive	Predicted negative		
Subtype	# analyzed	(exact match)	(mismatches)	(exact match)	(mismatches)		
G	8	8	0	8	0		

Positive results are those having exact matches with both primers and the probe; negative results show one or more mismatches in either the primers or probe. Numbers in parentheses () are instances where the mismatches were so great that the sequences were absent from the analysis results.

In summary, two of the three *bont*/A assay designs (CbA 2.323 and CbA 4.16566) were experimentally shown to be sensitive and specific for target *bont*/A1-A4 genes when tested in the laboratory. However, assay CbA 2.341 showed several nonspecific results. Subsequent *in silico* analysis of the assays indicated all three assays were capable of identifying genes from additional BoNT/A1-A4 strains, as well as BoNT/A5-A8 subtypes, with the sole exception of *bont*/A8 with the CbA 4.16566 assay.

## Results for *bont*/B assays

### Assay design

The *bont/*B genes show greater conservation in nucleotide sequence between subtypes than other serotypes, with differences ranging from 0.8 to 4.0% (Table 3B). However, even single nucleotide sequence differences among subtype variants may affect primer or probe matches, resulting in false negatives/detection failures. Subtypes BoNT/B2, BoNT/B3, and BoNT/B6 are closely related, with less than 1.2% difference between subtype genes and, interestingly, identical BoNT/B1, /B2, and /B6 toxins are expressed by both *C. botulinum* and *C. sporogenes* strains. In addition, the BoNT/B serotype also contains a unique *bont/*B gene that produces a protein that is not toxic. Generally, a lack of neurotoxicity is due to a complete absence of a toxin gene, but this BoNT/B5 variant contains a gene having a G-T mutation that introduces a stop codon at amino acid 128. This mutation produces a severely truncated, nontoxic protein [BoNT/(B)]. Currently, this *bont/*(B) gene has only been detected in combination with a *bont/*A gene, so that these strains are neurotoxigenic, producing only BoNT/A1.

The assay designs for *bont*/B-containing strains were developed using 48 DNA sequences from *bont*/B1–B6 to *bont*/(B) genes. Two assay designs based on primer/probe sets CbB 2.11 and CbB 2.295 (Table 4, *bont*/B), were tested in the laboratory for sensitivity and specificity, and subsequently evaluated for potential binding to genes from BoNT/B1–B8 subtypes using *in silico* analysis. A third assay based on primer/probe set Cb(B)stop was designed specifically to discriminate between the gene encoding *bont*/B5 and the *bont/*(B) gene. The *bont/*(B) assay based on primer/probe set Cb(B)stop contains two probes with distinct dyes: the FAM dye is specific for the detection of the *bont*/(B) gene and the VIC dye detects *bont*/B genes encoding active BoNT/B toxins. The assay design for *bont*/(B)-containing strains was developed using 11 DNA sequences from *bont*/(B) genes. This assay was also laboratory tested for sensitivity and specificity, and subsequently evaluated for potential binding to genes from additional BoNT/B1-B8 subtypes using *in silico* analysis.

### RT-PCR sensitivity testing

Two assays (CbB 2.11 and CbB 2.295) were tested in experiments with DNA preparations from 33 BoNT/B strains that included DNA sequences from BoNT/B1, BoNT/B2, BoNT/B4, and BoNT/B5 including bivalent BoNT/A2B5, BoNT/B5A4, and BoNT/A1(B) subtypes. The Cb(B) stop assay was tested in experiments using five BoNTA1(B) strains. The results, summarized in Table 5 and detailed in Supplementary Table S6B, indicate that all assays successfully detected their target *bont/*B1, *bont/*B2, *bont/*B4, and *bont/*B5-containing strains, including bivalent *bont*/B and *bont*/(B) genes. The assay based on primer/probe sets Cb(B) stop was tested using DNA from bivalent strains and *bont*/(B) gene sequences from five BoNT/A1(B) strains. Sensitivity testing for all assays was done using 1 pg. of target DNA per assay.

### RT-PCR specificity testing

Specificity testing results showed that two of the three *bont*/B assays [CbB 2.11 and Cb(B) stop] did not amplify DNA from nontarget *bont*-containing strains, other clostridial species, or additional prokaryotic and eukaryotic species when 100 pg. of DNA were used. The CbB 2.295 assay resulted in one nonspecific positive with DNA from BoNT/F6 Eklund 202F, with a Ct value of 38.4 (Table 5). It is known that the BoNT/F6 Eklund 202F genome contains remnants of an ancestral *bont*/B gene, but nucleotide analysis indicates that the *bont*/B fragments are not in the area encompassed by the CbB 2.295 primer/probe set (Carter et al., 2013). Results from specificity testing of the Cb(B) stop assay indicates that this assay can detect and distinguish both *bont/*(B) and *bont/*B genes from all tested subtypes. Positive controls for the three assays included DNA from: BoNT/B1 VPI 3,801, BoNT/A1(B) Hall 3676 and BoNT/B5A4 strain 657.

### *In silico* evaluation of additional BoNT/B subtypes

The primer/probe sequences for the three *bont/*B gene assays [CbB 2.11, CbB 2.295, and Cb(B) stop] are all located within the 5′ *bont/B* gene nucleotide region between bp 341–499, so that primers and probes overlap. Interestingly the *bont/*B genes from four out of five BoNT/B2 strains isolated in Argentina contain one (C→T) mutation at base pair 374 that results in a single mismatch in the forward primers of CbB 2.11, and CbB 2.295, and in the probe sequence of Cb(B) stop.

**Figure 1:**
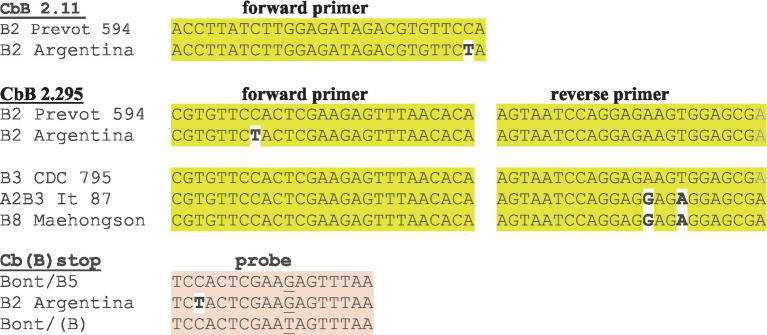
Mismatches in forward and reverse primers and probes that predict a likely reduction in RT-PCR detection sensitivity with *bont/*B assays. The *bont/*B variant gene from Argentinean BoNT/B2 strains shows mismatches in the forward primer or probe from each of the three *bont/*B assays.

The *in silico* analysis predicted that the assays based on primer sets CbB 2.11 and Cb(B) stop were capable of detecting *bont*/B1-/B8 subtypes, having exact matches against the forward and reverse primers and the probes, with the exception of the above mentioned Argentinean *bont/*B2 genes (Table 6B; Figure 1). However, *bont*/B mutations in specific subtype variants resulted in several mismatches with the primers of CbB 2.295 including the Argentinean *bont/*B2 gene mismatch, and two mismatches (450A→G and 453 T→A) with both the *bont/*B3 gene from BoNT/A2B3 It 87 and *bont/*B8 genes (Figure 1). With the exception of *bont/*B8, these mismatches are not subtype-specific, but are mutations that represent individual subtype variant differences.

In summary, the three assays were shown to be sensitive and specific for the detection of *bont*/B1, *bont*/B2, *bont*/B4, and *bont*/B5 genes, including *bont*/(B), when tested in the laboratory. Subsequent *in silico* analysis of the assays indicated two assays, CbB 2.11 and Cb(B) stop, were capable of identifying genes from additional BoNT/B1-B8 subtypes as well, with the lone exception of *bont/*B2 genes from strains originating in Argentina. The mismatch that occurred in the MGB probe of Cb(B)stop for the Argentinean BoNT/B2 strains will likely result in detection failure (due to the single base mismatch sensitivity of MGB probes) and the mismatches in the CbB 2.11 and CbB 2.295 primers may also result in detection failures. To correct the Argentinean *bont*/B2 mismatch, new degenerate primers and probes can be designed incorporating a degenerate Y nucleotide, (=C or T) nucleotides at the affected site, resulting in exact matches for all *bont*/B2 genes. Interestingly, mismatches that have occurred with all of the primer/probe sets have been associated not with subtype differences, but rather within variants of the same toxin subtype, with the exception of the three known *bont/*B8 genes. However, the single addition of a degenerate Y nucleotide in the CbB 2.11 and Cb(B) stop assays would enable detection of all *bont/*B genes and differentiation between *bont/*B and *bont/*(B) using RT-PCR.

## Results for *bont/*C assays

### Assay design

In addition to the classic BoNT/C subtype there is a chimeric subtype (BoNT/CD) where the 5′ region of the gene matches the *bont/*C gene and the 3′ region matches the *bont/*D gene. Thus, assays that are specific for only BoNT/C subtypes must be carefully designed. These assay designs were developed using 12 DNA sequences from both *bont/*C and *bont/*CD genes. Two assay designs based on primer/probe sets CbC 2.11 and CbC 2.968 (Table 4, *bont*/C), were tested in the laboratory for sensitivity and specificity, and genes from additional BoNT/C and BoNT/CD strains were subsequently evaluated using *in silico* analysis.

### RT-PCR sensitivity testing

The two *bo*nt/C assays using CbC 2.11 and CbC 2.968 primer/probe sets were experimentally tested in triplicate with a target panel of 10 serotype C or C/D strains. The results show that the assays detect both *bont/*C and the chimeric *bont/*CD genes when 1 pg. of DNA was used (Table 5; Supplementary Table S6C).

### RT-PCR specificity testing

Specificity testing results showed that the assay based on the CbC 2.11 primer/probe set did not amplify DNA from nontarget *bont*-containing strains (Table 5), other clostridial species, or additional prokaryotic and eukaryotic species when 100 pg. of DNA were used. While the assay using the CbC 2.968 primer/probe set sensitively detected all *bont*/C and *bont*/CD genes, it also exhibited detection in DNA preparations from multiple non-neurotoxigenic clostridial strains, including *C. perfringens*, *C. sporogenes*, and *C. tetani*, at high Ct values ranging from 38 to 39.8. The positive control for the specificity testing was DNA from one of the two BoNT/C Copenhagen 41/59–60 subclones.

### *In silico* evaluation of additional BoNT/C subtypes

*In silico* analysis indicated that the primers and probes associated with CbC 2.11 and CbC 2.968 were exact matches for the detection of genes encoding both BoNT/C and BoNT/CD (Table 6C). Degenerate primers were added to ensure that both assays were able to detect both *bont*/C and *bont*/CD genes. With the CbC 2.11 assay, only one degenerate nucleotide was needed, but with the CbC 2.968 assay, four degenerate nucleotides were incorporated into the primers and probe. This relatively high number of degenerate nucleotides may have contributed to the nonspecific positive detections seen during specificity testing and, importantly, the degenerate nucleotides are necessary for the specific detection of *bont*/CD genes. Thus, if the degenerate nucleotides in CbC 2.968 were converted to non-degenerate nucleotides (TTTACGA**G**ATCAATTGAGGA**G**GCTTTGG, ACTTACTTTCCTA**C**ACTAGCT, and AAAGTAAAT**G**CGGGTGTTCAAGGTGG), it is predicted that the CbC 2.968 assay would exclusively detect *bont*/C genes, while the CbC 2.11 assay would be specific for both *bont*/C and *bont*/CD (plus *bont*/CDDC). This change would provide for differentiation between *bont*/C and *bont*/CD genes, which could be useful in diagnosing animal botulism cases. The use of concurrent assays would provide specificity for *bont*/C (both CbC 2.11 and CbC 2.968 should be positive) and *bont*/CD (only CbC 2.11 should be positive), with a negative result for CbC 2.11 and a positive result with only CbC 2.968 signaling a nonspecific positive. An examination of the primers and probe sequences associated with CbC 2.968 showed that portions (~60–90% coverage) of primer/probe sequences were exact matches with a wide variety of bacteria, including various *Clostridium* species, *Clostridioides difficile*, and some *Streptococcus* and *Weissela* species. Removal of the degenerate nucleotides might also minimize these nonspecific results.

In summary, the CbC 2.11 assay passed all laboratory sensitivity and specificity testing and is useful for detecting *bont/*C and *bont/*CD genes using RT-PCR. The CbC 2.968 assay is also positive for both *bont*/C and *bont*/CD but it also showed some nonspecific positives. However, alteration of the CbC 2.968 primers and probe by replacing degenerate nucleotides with non-degenerate nucleotides could minimize nonspecific reactions and also provide for differentiation between *bont*/C and *bont*/CD genes using both assays concurrently.

## Results for *bont/*D assays

### Assay design

As with the BoNT/C serotype, the BoNT/D serotype is composed of the classic BoNT/D subtype and a chimeric subtype, BoNT/DC, where the 5′ region of the gene matches the *bont/*D gene and the 3′ region matches the *bont/C* gene. To ensure assay specificity, the *bont/*D and *bont/DC* assay designs were developed using DNA sequences from both *bont/*D (6 sequences) and *bont/*DC genes (4 sequences). Two assay designs based on primer/probe sets CbD 2.0 and CbD 2.276 (Table 4, *bont*/D), were tested in the laboratory for sensitivity and specificity, and genes from additional BoNT/D and BoNT/DC strains were subsequently evaluated using *in silico* analysis.

### RT-PCR sensitivity testing

Serotype D is relatively rare, so the two *bont*/D assay designs, CbD 2.0 and CbD 2.276, were experimentally tested with a target panel of only three serotype D strains. The two assays detected the target *bont*/D genes from these strains when 1 pg. of DNA was tested (Table 5; Supplementary Table S6D), however, no strains containing *bont/*DC genes were experimentally tested.

### RT-PCR specificity testing

Specificity testing results showed that the *bont*/D assays did not amplify DNA from nontarget *bont*-containing strains (Table 5), other clostridial species, or additional prokaryotic and eukaryotic species when 100 pg. of DNA were used. The positive control used was DNA from BoNT/D strain 1875.

### *In silico* evaluation of additional BoNT/D subtypes

The *in silico* analysis results indicated that the assay primers and probes for the CbD 2.0 and CbD 2.276 assays are an exact match for the detection of both *bont/*D and *bont/*DC genes (Table 6D). As both assays are specific for the botulinum neurotoxin light chain domain, which is conserved between *bont*/D and *bont*/DC genes, they cannot be used to discriminate between these two subtypes.

In summary, both *bont*/D assay designs were shown to be sensitive and specific for target *bont*/D genes when tested in the laboratory, and subsequent *in silico* analysis of the assays indicated them to be capable of identifying genes from BoNT/D and BoNT/DC subtypes.

## Results for *bont/*E assays

### Assay design

While the *bont*/E gene sequences of BoNT/E subtypes are less variable than BoNT/F or BoNT/A subtypes, there are more subtypes (12 total) (Table 3, *bont*/E), including the closely related BoNT/E1/E2/E3 subtypes, the *C. butyricum* BoNT*/*E4 and BoNT/E5 subtypes, and the divergent BoNT/E9, from Argentina. The assay designs for *bont*/E-containing strains were developed using 24 DNA sequences from *bont*/E1-/E6 genes. Two assay designs based on primer/probe sets CbE 2.0 and CbE 2.693 (Table 4E), were tested in the laboratory for sensitivity and specificity, and subsequently evaluated for potential binding to genes from additional BoNT/E1-E12 subtypes and variants using *in silico* analysis.

### RT-PCR sensitivity testing

Two assay designs, CbE 2.0 and CbE 2.693, were experimentally tested with DNA from BoNT/E1-/E4 strains. The results (Table 5; Supplementary Table S6E) show that the CbE 2.0 assay successfully detected DNA from the target strains. Unlike CbE 2.0, the CbE 2.693 assay yielded results that were variable and less sensitive, with a complete failure to detect *bont* genes from one subtype variant (BoNT/E1 ATCC 17852), and variable results with several other DNA preparations including detection failures and Ct values of 36.1–39.8 when 1 pg. of target DNA was used in testing.

### RT-PCR specificity testing

Specificity testing results showed that the *bont*/E assays did not amplify DNA from alternative *bont*-containing strains (Table 5), other clostridial species, or additional prokaryotic and eukaryotic species when 100 pg. of DNA were used. Positive controls for the specificity assays included *bont/*E1 (ATCC 17852) and *bont/*E3 (CDC 5258) DNA. While sensitivity testing of DNA from BoNT/E1 ATCC 17852 with CbE 2.693 resulted in a failure to detect the *bont/*E1 gene, a positive result (Ct 29.9) was observed when this DNA preparation was tested at 100 pg. DNA (Supplementary Table S6E). Thus, the variable results seen with CbE 2.693 appear to be a sensitivity issue that is not related to specificity.

### *In silico* evaluation of additional BoNT/E subtypes

**Figure 2:**
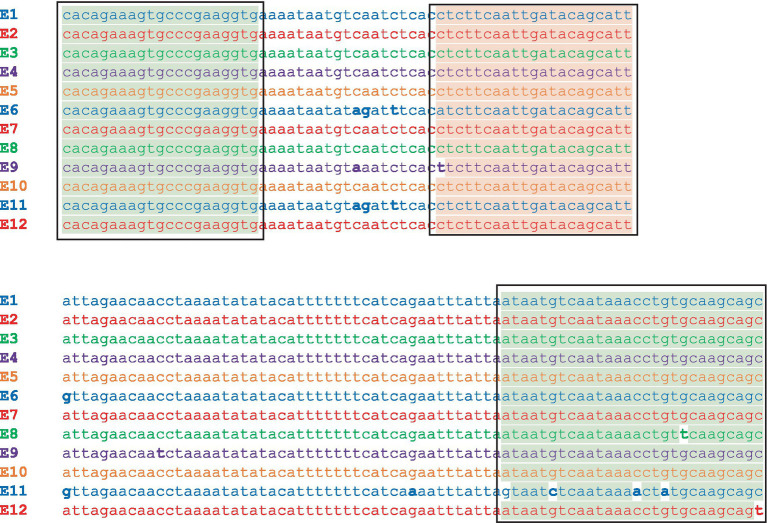
Sequence alignment of the gene fragment in the 12 *bont*/E subtypes between the CbE 2.693 forward and reverse primers. The alignment shows exact sequence matches in subtypes *bont/*E1-E7, with a single mismatch in the probe for *bont/*E9 and mismatches in the reverse primer for *bont/*E8, *bont/*E11, and *bont*/E12. The primer sites are highlighted in green and the probe site is highlighted in red.

*In silico* analysis results indicated that the CbE 2.0 primers and probe are a perfect match for the detection of 11/12 BoNT/E subtypes (Table 6E), with a single G→T mismatch in the probe for the assay with the unique *bont*/E8 Bac-02-06430 gene. Addition of the degenerate nucleotide K (G or T) in place of G (TGATTCTAATGGAACAAGT**K**A) would enable CbE 2.0 capable of identifying genes from all BoNT/E subtypes. CbE 2.693 probe had one mismatch with *bont/*E9 and the reverse primer showed mismatches with *bont/*E8, *bont/*E11, and *bont/*E12 (Figure 2).

In summary, the CbE 2.0 assay was shown to be sensitive and specific for target *bont*/E genes when tested in the laboratory. The CbE 2.693 assay was less sensitive than CbE 2.0 but showed good results when 100 pg. of *bont/*E1 or *bont/*E4 DNA was used for testing. Subsequent *in silico* analysis of the assays indicated that the CbE 2.0 assay should be capable of identifying genes from BoNT/E1–E7 to E9–E12 subtypes, while the CbE 2.693 assay appears to be limited to detection of *bont/*E1-E7 and *bont/*E10. Addition of a single degenerate nucleotide in the probe for CbE 2.0 should render it capable of detecting genes from all BoNT/E subtypes and subtype variants.

## Results for *bont/*F assays

### Assay design

The BoNT/F serotype is highly variable; subtypes can differ by more than 23% in DNA sequence. The more divergent subtypes are *C. baratii* BoNT/F7, and Argentinean BoNT/F5 (Table 3, *bont*/F). BoNT/F5 is a chimeric toxin having a unique enzymatic domain with a DNA sequence that differs by more than 30% from all other toxin subtypes. In addition, the BoNT/F subtypes show a high degree of identity with BoNT/E, with as little as 25% difference in DNA sequence between them. The extreme diversity within this serotype and its close relationship with type E presented challenges when designing assays that specifically detect genes from all BoNT/F subtypes, and the diversity among the subtypes required customized assay designs for *bont/*F5 and *bont/*F7 genes. Thus, the CbF 2.0 primer/probe set was developed using DNA sequences from *bont*/F1 (7), *bont*//F2 (2), *bont*//F3 (1), *bont*//F4 (2), and *bont*//F6 (2) genes, while CbF 2.1084 was specifically developed to detect *bont/*F5 (with two sequences) and CbF 2.161 was developed to detect *bont/*F7 (with three sequences). Three assay designs, based on primer/probe sets CbF 2.0, CbF 2.161, and CbF 2.1084 (Table 4F), were experimentally tested for sensitivity and specificity, and subsequently computationally evaluated for potential binding to genes from additional BoNT/F1-F9 subtypes.

### RT-PCR sensitivity testing

The three assays were experimentally tested in triplicate with eight DNA preparations from BoNT/F1, /F2, /F6, and /F7 strains. The CbF 2.0 assay detected *bont/*F1, *bont/*F2, and *bont/*F6, while the CbF 2.1084 assay was shown to be positive only for *bont/*F2 (with CbF 2.0), and CbF 2.161 is specific only for *bont/*F7 (Table 5; Supplementary Table S6F). The three assays detected the target when 1 pg. of DNA was tested.

### RT-PCR specificity testing

Specificity testing results showed that the *bont*/F assays did not amplify DNA from alternative *bont*-containing strains (Table 5), including those containing *bont/E* genes, other clostridial species, or additional prokaryotic and eukaryotic species when 100 pg. of DNA were used. Positive controls for the specificity assays were: *bont/*F1 (CDC 2821) and *bont/*F2 (An436) DNA for assay CbF 2.0, *bont/*F2 DNA for assay CbF 2.1084, and *bont/*F7 (Sullivan) DNA for assay CbF 2.161.

### *In silico* evaluation of additional BoNT/F subtypes

The *in silico* analysis results predict that all *bont/F* subtype genes can be detected using a combination of the three assays (Tables 5, 6F). The CbF 2.0 primers/probes are an exact match for *bont/*F1-F4, *bont/*F6, and *bont/*F8-F9, while the CbF 2.1084 primers/probes match *bont/*F2, *bont/*F3, and *bont/*F5, and CbF 2.161 is a specific match for only *bont/*F7 (Table 7). While all BoNT/F subtype strains are rare, the most common subtype detected in the United States is BoNT/F7, in *C. baratii* strains.

**Table 7:** Predicted positive results of the designed assays with the *bont*/F1-9 genes from all BoNT/F subtypes.

	CbF 2.0	CbF 2.161	CbF 2.1084
*bont/*F1	X		
*bont/*F2	X		X
*bont/*F3	X		X
*bont*/F4	X		
*bont/*F5			X
*bont/*F6	X		
*bont/*F7		X	
*bont/*F8	X		
*bont/*F9	X		

Predictions are based on exact primer/probe matches for each subtype sequence.

In summary, the three *bont*/F assays were designed to be sensitive and specific for all known *bont*/F genes, and laboratory testing confirmed their utility for detecting *bont*/F1, /F2, /F6, and /F7. Subsequent *in silico* analysis of the assays predicted them to be capable of identifying genes from additional BoNT/F1-F9 subtypes. This indicates that the three assays, in combination, may be useful for detecting *bont/*F genes and differentiating *bont/*F5 and *bont/*F7 genes from other subtypes using RT-PCR.

## Results for *bont/*G assay

### Assay design

BoNT/G strains are rare, with three strains being isolated from Argentina and five strains from Switzerland. The gene sequences from these BoNT/G strains are homogeneous, with no toxin subtypes or variants noted, regardless of isolation location. Two assay designs, based on primer/probe sets CbG 2.0 and CbG 2.83 (Table 4, *bont*/G), were tested in the laboratory for sensitivity and specificity, and subsequently evaluated for potential binding to additional *bont/*G DNA sequences using *in silico* analysis.

### RT-PCR sensitivity testing

The two assays were tested in triplicate experiments with a target panel of six serotype G strains (Supplementary Table S6G). The CbG 2.83 was found to be more sensitive than CbG 2.0, which failed to detect *bont/*G when 1 pg. of DNA was used but detected the CDC 2741 *bont/*G gene with a Ct of 28.3 when 100 pg. of DNA were tested (Table 5; Supplementary Table S6G).

### RT-PCR specificity testing

Specificity testing results showed that the *bont*/G assays did not detect DNA from nontarget *bont*-containing strains (Table 5), other clostridial species, or additional prokaryotic and eukaryotic species when 100 pg. of DNA were used. The positive control was DNA from *C. argentinense* BoNT/G strain CDC 2741.

### *In silico* evaluation of additional BoNT/G strains

*In silico* analysis results indicated that both assay primers and probes are an exact match for the detection of all available *bont/G* gene sequences (Table 6G).

In summary, both *bont/*G assays were found to be specific for target *bont/*G genes. Of the two assays, CbG 2.83 was shown to be more sensitive than CbG 2.0. Testing with DNA containing *bont* genes from other serotypes, DNA from other clostridial species, and DNA from multiple prokaryotic and eukaryotic species produced negative results. Subsequent *in silico* analysis of the assays indicated the two assays were capable of identifying genes from additional BoNT/G strains. Both assays are useful for detecting *bont/G* genes using RT-PCR.

### Results of *in silico* evaluation of ISO 17919:2013 assays

A total of 13 individual primer/probe sets were evaluated for their predicted ability to detect genes from the currently identified eight BoNT/A subtypes, eight BoNT/B subtypes, 12 BoNT/E subtypes, and nine BoNT/F subtypes. These subtypes contained representatives that were expressed by all seven BoNT-producing species (*C. botulinum*, *C. parabotulinum*, *C. sporogenes*, *C. novyi sensu lato*, *C. butyricum*, *C. baratii*, and *C. argentinense*). Results from the analyses are shown in Supplementary Table S7. During the analysis process, we noted that the CBMLE reverse primer contained a C (highlighted and underlined here—GCTATTGATCCAAAAC**C**GTGA), which was a universal mismatch to all bont/E genes with the exception of a single sequence, X62089. X62089 was an early sequence of BoNT/E1 Beluga and comparison of this sequence with four other BoNT/E1 Beluga sequences revealed that the C nucleotide was unique to that particular sequence. As all other Beluga sequences contained a G at that site. It is likely that the CBMLE primers were designed using X62089.

In many cases, predicted assay failures were due to lack of identification of newly identified toxin subtypes, for example *bont*/B8, *bont*/E8, *bont*/E9, *bont*/E11, *bont*/E12, *bont*/F7, and *bont*/F8. With the exception of *bont*/F7, the addition of one or two degenerate nucleotides to the primers or probes should provide for exact matches using at least one of the ISO assays. It is important to note that primer/probe mismatches may not be the definitive factor in these assays, and that additional laboratory testing using purified DNA representing each of the BoNT subtypes is needed to verify these predictions.

## Discussion

Identification of the specific botulinum neurotoxin involved in a botulism case can involve either the identification of the toxin protein or the toxin gene, or both. Historically, the toxin protein has been the target of assays using various techniques such as ELISA, Endo-PEP mass spectrometry, or antigen neutralizations (mouse bioassays). DNA-based assays such as PCR and RT-PCR have recently been adopted in many laboratories to rapidly identify the toxin gene and compliment protein assay results. In addition to rapid detection of the presence of toxin genes, they also provide information as to toxin serotype and, in some cases, subtype. To have confidence in the DNA assay results and to avoid false positives or negatives, thorough design and testing must be performed.

The botulinum neurotoxins are an extremely diverse group of protein toxins, and this presents challenges when designing RT-PCR assays, where a single nucleotide mismatch in either the forward or reverse primer or the probe sequence may be the difference between successful detection and failure to detect the toxin gene. As more *bont* gene diversity is being discovered and access to the strains containing these genes is sometimes difficult or impossible, continuing evaluations using *in silico* analysis of the assays is imperative.

These RT-PCR assays were computationally designed using 150 *bont* gene sequences representing 21 different BoNT subtypes plus the *bont*/(B) variant of *bont*/B5. Seventeen assays were selected for laboratory testing for assay sensitivity and specificity using 82 DNA preparations containing *bont* genes; 30 clostridial species that do not contain *bont* genes; 110 additional bacterial species; and 37 eukaryotic species, including yeast, fungi, protozoans, plants, and animals. Results from testing revealed these assays were generally sensitive and specific, and identified sensitivity or specificity issues. Subsequent *in silico* evaluation of these assays with *bont* gene sequences from newer BoNT subtypes predicted the assays will be successful in detecting these genes with rare exceptions.

Experimental testing has revealed these RT-PCR assays to be sensitive for the detection of target *bont* genes, with two exceptions. The CbE 2.693 assay produced variable results when tested in triplicate against preparations containing 1 pg. of DNA from *bont/*E genes representing BoNT/E1-E4 subtypes. However, this assay successfully detected *bont/*E1 and *bont/*E4 genes when 100 pg. of DNA was used for testing. In addition, the CbG 2.0 assay failed to detect *bont/*G genes when 1 pg. of DNA were used but was successful when testing was done using 100 pg. DNA.

Rigorous specificity testing using DNA preparations containing genes representing non-target BoNT subtypes, other clostridial species, and a range of prokaryotic and eukaryotic species has shown these assays to be specific for target genes, with a few exceptions. The CbA 2.341 assay was a notable exception where, in addition to sensitively detecting *bont* genes from BoNT/A1-A5 subtypes, also yielded false positive results with DNA preparations representing several *bont/*B, *bont/*C, *bont/*D, *bont/*E, and *bont/*F subtypes. The CbB 2.295 assay also showed one instance of detection of DNA from a non-target gene (*bont/*E6 from Eklund 202F). The CbC 2.968 assay was interesting in that it showed positive results against DNA preparations from eight distinct clostridial species that did not contain *bont* genes, including *C. perfringens*, *C. novyi*, and *C. tetani*.

*In silico* assessments of the abilities of these assays to detect *bont* genes from all known subtypes, conducted between November 2022 and February 2024, predicts that one or more assays for each serotype will be effective for the detection of all toxin subtypes, with two exceptions. Single nucleotide mismatches in the *bont/*B gene from BoNT/B2 strains originating in Argentina and the *bont*/E gene from BoNT/E8 Bac-02-06430 are responsible for potential failures to detect these genes with any of the three *bont/*B or the two *bont*/E assays, illustrating the impact of individual nucleotide mutations on PCR assays. The use of degenerate nucleotides in these locations should allow for detection of genes from all BoNT/B and BoNT/E strains.

On the basis of laboratory testing and *in silico* analysis, it appears that the original panel of 17 designed assays could be reduced to 10 total for routine testing covering all BoNT serotypes; however, further testing using DNA representing newer toxin subtypes should be done to verify these *in silico* results. In addition, testing of spiked or naturally contaminated samples will be necessary to assure the assays will function in a variety of matrices.

A series of *in silico* analyses were also performed to compare the potential ability to detect genes from all BoNT subtypes with these assays versus those listed in the ISO/TC 17919:2013. The ISO assays were developed and tested from 2001 to 2009, and additional assessments were done in 2013. However, many new subtypes and subtype variants have been identified since then, so an updated *in silico* analysis was done as part of this study. The assessment predicted potential failures to detect genes from eight toxin subtypes. However, addition of degenerate nucleotides at one or two locations within the primers should improve performance of these assays, resulting in successful detection of genes from all toxin subtypes except BoNT/F7.

In summary, this set of RT-PCR assays, which were computationally designed and evaluated both experimentally and with additional computational testing, are meant to provide laboratories with a suite of assays that detect *bont* genes from all known subtypes of the seven different BoNT serotypes. The RT-PCR *bont* assays utilize readily available reagents and equipment that can be used for rapid detection within clinical, food, or environmental samples worldwide. Different strategies for the use of these assays and interpretation of the results can be tailored for individual laboratory needs and goals. For example, a foodborne botulism panel could be developed using six assays [CbA 2.323, Cb(B) stop, CbE 2.0, and all three *bont*/F assays]. Use of this panel would not only identify genes from the known agents of foodborne botulism but could also differentiate between active *bont/*B5 producers and strains containing silent (B) genes, plus differentiate *bont*/F5 and *bont/*F7 genes from other *bont*/F subtypes. The ability to differentiate these subtypes enables rapid identification of *C. baratii bont*/F7, which is an emerging public health issue. A panel of 3–5 assays focused on detection of animal botulism cases could also be developed using CbC 2.11, CbC 2.968, CbD 2.0, or CbD 2.276, and the possible addition of *bont*/A and *bont*/B assays, depending on the specific animals involved (poultry vs. horses, for example). Importantly, these assays could complement other methods that identify the presence of the BoNT protein(s) and provide rapid results where decisions for public health and/or therapeutic intervention may be critical.

## Data availability statement

The original contributions presented in the study are included in the article/Supplementary material, further inquiries can be directed to the corresponding author.

## Author contributions

SP: Conceptualization, Formal analysis, Methodology, Project administration, Resources, Supervision, Writing – original draft, Writing – review & editing. KH: Data curation, Formal analysis, Funding acquisition, Investigation, Methodology, Validation, Writing – original draft, Writing – review & editing. JG: Data curation, Formal analysis, Methodology, Project administration, Software, Validation, Writing – original draft, Writing – review & editing. TS: Data curation, Formal analysis, Methodology, Validation, Writing – original draft, Writing – review & editing.

## References

[ref1] Akbulut D. Grant K. A. McLauchlin J. (2004). Application and development of real-time PCR assays to detect fragments of the *Clostridium botulinum* types a, B, and E neurotoxin genes for investigation of human food-borne and infant botulism. Foodborne Pathog. Dis. 1, 247–257. doi: 10.1089/fpd.2004.1.247 15992287

[ref3] Anon (2013). Botulinum toxin gene real-time PCR assay laboratory response network (LRN).

[ref4] Anon (2021). ISO/TC 17919:2013 microbiology of the food chain—Polymerase chain reaction (PCR) for the detection of food-borne pathogens—Detection of botulinum type a, B, E and F neurotoxin-producing clostridia. International Standard 2021.

[ref5] Braconnier A. Broussolle V. Perelle S. Fach P. Nguyen-The C. Carlin F. (2001). Screening for *Clostridium botulinum* type a, B, and E in cooked chilled foods containing vegetables and raw material using polymerase chain reaction and molecular probes. J. Food Prot. 64, 201–207. doi: 10.4315/0362-028x-64.2.201, PMID: 11271768

[ref6] Carter A. T. Stringer S. C. Webb M. D. Peck M. W. (2013). The type F6 neurotoxin gene cluster locus of group II *Clostridium botulinum* has evolved by successive disruption of two different ancestral precursors. Genome Biol. Evol. 5, 1032–1037. doi: 10.1093/gbe/evt068 23645598 PMC3673618

[ref7] De Medici D. Anniballi F. Wyatt G. M. Lindstrom M. Messelhausser U. Aldus C. F.. (2009). Multiplex PCR for detection of botulinum neurotoxin-producing clostridia in clinical, food, and environmental samples. Appl. Environ. Microbiol. 75, 6457–6461. doi: 10.1128/AEM.00805-09, PMID: 19684163 PMC2765140

[ref8] Fach P. Fenicia L. Knutsson R. Wielinga P. R. Anniballi F. Delibato E.. (2011). An innovative molecular detection tool for tracking and tracing *Clostridium botulinum* types a, B, E, F and other botulinum neurotoxin producing Clostridia based on the GeneDisc cycler. Int. J. Food Microbiol. 145, S145–S151. doi: 10.1016/j.ijfoodmicro.2010.04.006, PMID: 20471128

[ref9] Fach P. Micheau P. Mazuet C. Perelle S. Popoff M. (2009). Development of real-time PCR tests for detecting botulinum neurotoxins a, B, E, F producing *Clostridium botulinum*, *Clostridium baratii* and *Clostridium butyricum*. J. Appl. Microbiol. 107, 465–473. doi: 10.1111/j.1365-2672.2009.04215.x 19291235

[ref10] Fenicia L. Fach P. van Rotterdam B. J. Anniballi F. Segerman B. Auricchio B.. (2011). Towards an international standard for detection and typing botulinum neurotoxin-producing Clostridia types a, B, E and F in food, feed and environmental samples: a European ring trial study to evaluate a real-time PCR assay. Int. J. Food Microbiol. 145, S152–S157. doi: 10.1016/j.ijfoodmicro.2011.02.001, PMID: 21353718

[ref11] Fernandes J. D. Hinrichs A. S. Clawson H. Gonzalez J. N. Lee B. T. Nassar L. R.. (2020). The UCSC SARS-CoV-2 genome browser. Nat. Genet. 52, 991–998. doi: 10.1038/s41588-020-0700-8, PMID: 32908258 PMC8016453

[ref12] Franciosa G. Ferreira J. L. Hatheway C. L. (1994). Detection of type a, B, and E botulism neurotoxin genes in *Clostridium botulinum* and other *Clostridium* species by PCR: evidence of unexpressed type B toxin genes in type a toxigenic organisms. J. Clin. Microbiol. 32, 1911–1917. doi: 10.1128/jcm.32.8.1911-1917.1994, PMID: 7989542 PMC263902

[ref13] Gans J. D. Wolinsky M. (2008). Improved assay-dependent searching of nucleic acid sequence databases. Nucleic Acids Res. 36:e74. doi: 10.1093/nar/gkn301, PMID: 18515842 PMC2475610

[ref14] Grenda T. Grabczak M. Sieradzki Z. Kwiatek K. Pohorecka K. Skubida M.. (2018). *Clostridium botulinum* spores in polish honey samples. J. Vet. Sci. 19, 635–642. doi: 10.4142/jvs.2018.19.5.635, PMID: 29929360 PMC6167343

[ref15] Hielm S. Hyytia E. Ridell J. Korkeala H. (1996). Detection of *Clostridium botulinum* in fish and environmental samples using polymerase chain reaction. Int. J. Food Microbiol. 31, 357–365. doi: 10.1016/0168-1605(96)00984-1, PMID: 8880323

[ref16] Hill B. J. Skerry J. C. Smith T. J. Arnon S. S. Douek D. C. (2010). Universal and specific quantitative detection of botulinum neurotoxin genes. BMC Microbiol. 10:267. doi: 10.1186/1471-2180-10-267, PMID: 20961439 PMC2973968

[ref17] Hill K. K. Smith T. J. Helma C. H. Ticknor L. O. Foley B. T. Svensson R. T.. (2007). Genetic diversity among botulinum neurotoxin-producing clostridial strains. J. Bacteriol. 189, 818–832. doi: 10.1128/JB.01180-06, PMID: 17114256 PMC1797315

[ref18] Hossain M. A. M. Uddin S. M. K. Hashem A. Mamun M. A. Sagadevan S. Johan M. R. (2022). Advancements in detection approaches of severe acute respiratory syndrome coronavirus 2. Malays. J. Med. Sci. 29, 15–33. doi: 10.21315/mjms2022.29.6.3, PMID: 36818907 PMC9910375

[ref19] Hutson R. A. Zhou Y. Collins M. D. Johnson E. A. Hatheway C. L. Sugiyama H. (1996). Genetic characterization of *Clostridium botulinum* type a containing silent type B neurotoxin gene sequences. J. Biol. Chem. 271, 10786–10792. doi: 10.1074/jbc.271.18.10786 8631890

[ref20] Kirchner S. Kramer K. M. Schulze M. Pauly D. Jacob D. Gessler F.. (2010). Pentaplexed quantitative real-time PCR assay for the simultaneous detection and quantification of botulinum neurotoxin-producing clostridia in food and clinical samples. Appl. Environ. Microbiol. 76, 4387–4395. doi: 10.1128/AEM.02490-09, PMID: 20435756 PMC2897425

[ref21] Kutyavin I. V. Afonina I. A. Mills A. Gorn V. V. Lukhtanov E. A. Belousov E. S.. (2000). 3′-minor groove binder-DNA probes increase sequence specificity at PCR extension temperatures. Nucleic Acids Res. 28, 655–661. doi: 10.1093/nar/28.2.655, PMID: 10606668 PMC102528

[ref2] Lennette E. H. Balows A. Hausler W. J. Shadomy H. J. American Society for Microbiology. (1985) Manual of Clinical Microbiology. 4th ed. Washington, D.C.: American Society for Microbiology.

[ref22] Lindstrom M. Keto R. Markkula A. Nevas M. Hielm S. Korkeala H. (2001). Multiplex PCR assay for detection and identification of *Clostridium botulinum* types a, B, E, and F in food and fecal material. Appl. Environ. Microbiol. 67, 5694–5699. doi: 10.1128/AEM.67.12.5694-5699.2001, PMID: 11722924 PMC93361

[ref23] Liu C. M. Aziz M. Kachur S. Hsueh P. R. Huang Y. T. Keim P.. (2012). BactQuant: an enhanced broad-coverage bacterial quantitative real-time PCR assay. BMC Microbiol. 12:56. doi: 10.1186/1471-2180-12-56, PMID: 22510143 PMC3464140

[ref24] Maikanov B. Mustafina R. Auteleyeva L. Wisniewski J. Anusz K. Grenda T.. (2019). *Clostridium botulinum* and *Clostridium perfringens* occurrence in Kazakh honey samples. Toxins 11, 472–486. doi: 10.3390/toxins11080472, PMID: 31412583 PMC6723067

[ref25] Masters A. M. Palmer D. G. (2021). Confirmation of botulism diagnosis in Australian bird samples by ELISA and RT rtPCR. J. Vet. Diagn. Invest. 33, 684–694. doi: 10.1177/10406387211014486, PMID: 33955287 PMC8229820

[ref26] Needleman S. B. Wunsch C. D. (1970). A general method applicable to the search for similarities in the amino acid sequence of two proteins. J. Mol. Biol. 48, 443–453. doi: 10.1016/0022-2836(70)90057-4, PMID: 5420325

[ref27] Nguyen D. H. Nguyen T. T. Nguyen H. T. (2022). Investigation of botulism in free-range duck farming in the Mekong Delta, Vietnam. Open Vet. J. 12, 632–638. doi: 10.5455/OVJ.2022.v12.i5.7, PMID: 36589392 PMC9789775

[ref28] Park H. Y. Lee K. Jung S. C. Cho Y. S. (2022). Prevalent toxin types of *Clostridium botulinum* in south Korean cattle farms. Vet. Anim. Sci. 15:100239. doi: 10.1016/j.vas.2022.100239, PMID: 35243127 PMC8885797

[ref29] Peck M. W. Smith T. J. Anniballi F. Austin J. W. Bano L. Bradshaw M.. (2017). Historical perspectives and guidelines for botulinum neurotoxin subtype nomenclature. Toxins 9:38. doi: 10.3390/toxins9010038, PMID: 28106761 PMC5308270

[ref30] Prevot A. (1953). Rapport d’introduction du president du sous-comite *Clostridium* pour l’unification de la nonmenclature des types toxigeniques de *C. Botulinum*. (introductory report from the chair of the *Clostridium* subcommittee for the unification of the nomenclature of toxigenic types of *C. botulinum*). Int. Bull. Bacter. Nom. 3, 120–123. doi: 10.1099/0096266X-3-2-3-120

[ref31] Sachdeva A. Defibaugh-Chavez S. L. Day J. B. Zink D. Sharma S. K. (2010). Detection and confirmation of *Clostridium botulinum* in water used for cooling at a plant producing low-acid canned foods. Appl. Environ. Microbiol. 76, 7653–7657. doi: 10.1128/AEM.00820-10, PMID: 20889791 PMC2976179

[ref32] Shin N. R. Yoon S. Y. Shin J. H. Kim Y. J. Rhie G. E. Kim B. S.. (2007). Development of enrichment semi-nested PCR for *Clostridium botulinum* types a, B, E, and F and its application to Korean environmental samples. Mol. Cell 24, 329–337. doi: 10.1016/S1016-8478(23)07347-8, PMID: 18182847

[ref33] Skarin H. Hafstrom T. Westerberg J. Segerman B. (2011). *Clostridium botulinum* group III: a group with dual identity shaped by plasmids, phages and mobile elements. BMC Genomics 12:185. doi: 10.1186/1471-2164-12-185, PMID: 21486474 PMC3098183

[ref34] Skarin H. Lindgren Y. Jansson D. S. (2015). Investigations into an outbreak of botulism caused by *Clostridium botulinum* type C/D in laying hens. Avian Dis. 59, 335–340. doi: 10.1637/10861-051214-Case, PMID: 26473687

[ref35] Smith L. Holdeman L. (1968). The Pathogenic Anaerobic Bacteria. Charles C. Thomas: Springfield, Illinois.

[ref36] Smith T. J. Schill K. M. Williamson C. H. D. (2023). Navigating the complexities involving the identification of botulinum neurotoxins (BoNTs) and the taxonomy of BoNT-producing Clostridia. Toxins 15, 545–554. doi: 10.3390/toxins15090545, PMID: 37755971 PMC10535752

[ref37] Smith T. J. Tian R. Imanian B. Williamson C. H. D. Johnson S. L. Daligault H. E.. (2021a). Integration of complete plasmids containing Bont genes into chromosomes of *Clostridium parabotulinum*, *Clostridium sporogenes*, and *Clostridium argentinense*. Toxins 13, 473–485. doi: 10.3390/toxins13070473, PMID: 34357945 PMC8310154

[ref38] Smith T. J. Williamson C. H. D. Hill K. K. Johnson S. L. Xie G. Anniballi F.. (2021b). The distinctive evolution of orfX *Clostridium parabotulinum* strains and their botulinum neurotoxin type a and F gene clusters is influenced by environmental factors and gene interactions via mobile genetic elements. Front. Microbiol. 12:566908. doi: 10.3389/fmicb.2021.566908, PMID: 33716993 PMC7952441

[ref39] Smith T. Williamson C. Hill K. Sahl J. Keim P. (2018). Botulinum neurotoxin-producing bacteria. Isn't it time that we called a species a species? MBio 9, e01469–e01418. doi: 10.1128/mBio.01469-18 30254123 PMC6156192

[ref40] Smith T. J. Xie G. Williamson C. H. D. Hill K. K. Fernandez R. A. Sahl J. W.. (2020). Genomic characterization of newly completed genomes of botulinum neurotoxin-producing species from Argentina, Australia, and Africa. Genome Biol. Evol. 12, 229–242. doi: 10.1093/gbe/evaa043 32108238 PMC7144720

[ref41] Souillard R. Le Marechal C. Ballan V. Mahe F. Chemaly M. Le Bouquin S. (2017). A bovine botulism outbreak associated with a suspected cross-contamination from a poultry farm. Vet. Microbiol. 208, 212–216. doi: 10.1016/j.vetmic.2017.07.022, PMID: 28888640

[ref42] Souillard R. Woudstra C. Le Marechal C. Dia M. Bayon-Auboyer M. H. Chemaly M.. (2014). Investigation of *Clostridium botulinum* in commercial poultry farms in France between 2011 and 2013. Avian Pathol. 43, 458–464. doi: 10.1080/03079457.2014.957644, PMID: 25175400

[ref43] Sozhamannan S. Holland M. Y. Hall A. T. Negrón D. Ivancich M. Koehler J. W.. (2015). Evaluation of signature Erosion in Ebola virus due to genomic drift and its impact on the performance of diagnostic assays. Viruses 7, 3130–3154. doi: 10.3390/v7062763, PMID: 26090727 PMC4488730

[ref44] Suen J. C. Hatheway C. L. Steigerwalt A. G. Brenner D. J. (1988a). *Clostridium argentinense* sp. nov.: a genetically homogeneous group composed of all strains of *Clostridium botulinum* toxin type G and some nontoxigenic strains previously identified as *Clostridium subterminale* or *Clostridium hastiforme*. Int. J. Syst. Bacteriol. 38, 375–381. doi: 10.1099/00207713-38-4-375

[ref45] Suen J. C. Hatheway C. L. Steigerwalt A. G. Brenner D. J. (1988b). Genetic confirmation of identities of neurotoxigenic *Clostridium baratii* and *Clostridium butyricum* implicated as agents of infant botulism. J. Clin. Microbiol. 26, 2191–2192. doi: 10.1128/jcm.26.10.2191-2192.1988, PMID: 3183004 PMC266845

[ref46] Szabo E. A. Pemberton J. M. Desmarchelier P. M. (1993). Detection of the genes encoding botulinum neurotoxin types a to E by the polymerase chain reaction. Appl. Environ. Microbiol. 59, 3011–3020. doi: 10.1128/aem.59.9.3011-3020.1993, PMID: 8215372 PMC182400

[ref47] Takeshi K. Fujinaga Y. Inoue K. Nakajima H. Oguma K. Ueno T.. (1996). Simple method for detection of *Clostridium botulinum* type a to F neurotoxin genes by ploymerase chain reaction. Microbiol. Immunol. 40, 5–11. doi: 10.1111/j.1348-0421.1996.tb03310.x, PMID: 8871522

[ref48] Weigand M. R. Pena-Gonzalez A. Shirey T. B. Broeker R. G. Ishaq M. K. Konstantinidis K. T.. (2015). Implications of genome-based discrimination between *Clostridium botulinum* group I and *Clostridium sporogenes* strains for bacterial taxonomy. Appl. Environ. Microbiol. 81, 5420–5429. doi: 10.1128/AEM.01159-15, PMID: 26048939 PMC4510194

[ref49] Williamson C. H. Sahl J. W. Smith T. J. Xie G. Foley B. T. Smith L. A.. (2016). Comparative genomic analyses reveal broad diversity in botulinum-toxin-producing Clostridia. BMC Genomics 17:180. doi: 10.1186/s12864-016-2502-z, PMID: 26939550 PMC4778365

