# Supplementary material for: Real-time PCR assays that detect genes for botulinum neurotoxin A–G subtypes
Source: Front Microbiol. 2024 May 30;15:1382056. doi: 10.3389/fmicb.2024.1382056 (PMC11169944; doi:10.3389/fmicb.2024.1382056)
Supplement: Supplementary file 1 [file Table_8.DOCX]

**Table S1.** Sensitivity and specificity panel: *bont* gene-containing clostridia. The strains listed below were used for sensitivity and specificity testing with target strains containing the *bont* gene of interest.

| **Subtype** | **Strain** | **Source** |
| --- | --- | --- |
| A1 | Schantz | (probable Hall A subculture) |
| A1 | ATCC 3502 (Hall 174) | Canned peas/ California, 1922 |
| A1 | ATCC 17862  (Meyer 73) | Oats/horse outbreak, 1921 |
| A1 | ATCC 19397 | Lister Institute, 1934 |
| A1 | ATCC 25763  (CDC KA38) | ATCC type strain |
| A1 | CDC 1757 | Infant, Oregon, 1977 |
| A1 | Hall | unknown |
| A1 | Hall 5675 | Canned cauliflower/Colorado, 1932 |
| A1 | Prevot Ppois | Peas/1974 |
| A1 | ATCC 449 | unknown |
| A1 | Hall 11481 | Beets/Montana, 1933 |
| A1(B) | CDC 1744 | Infant, Pennsylvania, 1977 |
| A1(B) | Hall 183 | corn/Fort Collins, Colorado, 1922 |
| A1(B) | Hall 3676 | Human feces, November, 1929 |
| A1(B) | Hall 3685a | string beans/Pueblo, Colorado, 1929 |
| A1(B) | CDC 2357 | Stool, Colorado |
| A2 | FRI honey | Honey/infant, New Jersey |
| A2 | Kyoto-F | Infant, Japan, 1978 |
| A2B5 | CDC 1436 | Infant, Utah, 1977 |
| A3 | Loch Maree | Wild-duck paste/Scotland 1922 |
| B5A4 | CDC 657 | Infant, Texas, 1976 |
| B1 | NCTC 7273 (beans) | WHO standard |
| B1 | okra | Okra/Tennessee |
| B1 | CDC 1656 | Infant, Utah, 1977 |
| B1 | CDC 1758 | Infant, Pennsylvania, 1977 |
| B1 | ATCC 17783 | unknown |
| B1 | L. Ds. Smith | unknown |
| B1 | Hall 6517 | Peppers/New Mexico, 1932 |
| B1 | CDC 6252 | unknown |
| B2 | ATCC 17843 (B5) | unknown |
| B2 | 213B | Canned shallots/Italy, 1929 |
| B2 | CDC 1828 | Infant, Missouri, 1978 |
| B2 | Prevot 892 | unknown |
| B2 | Prevot 59 | unknown |
| B2 | Prevot 25NCASE | 1953 |
| B2 | Prevot 1740 | Ham/France, 1950 |
| B2 | Prevot B | Weinberg collection, 1941 |
| B2 | Prevot 314A | unknown |
| B2 | Smith L-590 | Bovine intestine |
| B2 | ATCC 8083 | Oats/equine outbreak, Sweden |
| B2 | CDC 6291 | unknown |
| B2 | Prevot 2345 | unknown |
| B5F2 | An436 | Infant, Sweden |
| B5F2 | CDC3281 | New Mexico, 1980 |
| B4 | Eklund 17B | Marine sediments/Washington, 1965 |
| B4 | ATCC 17844 | unknown |
| B4 | 100688 | unknown |
| C | Stockholm | Mink, Sweden, 1990 |
| C | 2048-Mich |  |
| C | ATCC 17849 | unknown |
| C | ATCC 17784 | unknown |
| CD | 003-9 | Japan |
| C | 468 | Continental Can Co., Illinois, 1971 |
| C | (Copenhagen) 41/59-60 | Cow liver/Denmark |
| CD | 6816 | Marine sediments/Washington, 1965 |
| C | Prevot 5714 | Animal botulism, France |
| C | Prevot 2260 | unknown |
| C | Copenhagen 41/59-60 | Cow liver/Denmark |
| D | 1873 | ham/Chad, 1958 |
| D | Schantz | (probable1973 subculture) |
| D | ATCC 11873 | unknown |
| E1 | ATCC 17852 | unknown |
| E1 | Beluga | Fermented whale flippers/US, 1952 |
| E1 | Prevot Ped1 | Sea mud/Greenland |
| E1 | Hazen 36208E (ATCC 9564) | Smoked salmon/Canada, 1932 |
| E2 | CDC 5247 | unknown |
| E2 | CDC 5906 | unknown |
| E3 | CDC 5258 | unknown |
| E3 | Prevot R81-3A | unknown |
| E4 | BL5262 | Infant, Rome, 1985 (*C. butyricum*) |
| F1 | CDC 2821 | crab |
| F1 | Langeland | Liver paste/Denmark, 1958 |
| F1 | 6/14 | (probable Langeland subculture) |
| F1 | Walls 8-G | Crabs/Virginia, 1966 |
| F6 | Eklund202F | Marine sediments/Pacific Coast, 1965 |
| F7 | Sullivan | Adult toxicoinfection, New York |
| G | CDC 2738 (SN 143/77) | autopsy specimen/Switzerland, 1978 |
| G | 1354 | unknown |
| G | CDC 2739 (GM 140/77) | autopsy specimen/Switzerland, 1978 |
| G | CDC 2740 (GM 73/78) | autopsy specimen/Switzerland, 1978 |
| G | CDC 2741 (GM 77/78) | autopsy specimen/Switzerland, 1978 |
| G | CDC 2742 (GM 56/78) | autopsy specimen/Switzerland, 1978 |
