# Supplementary material for: Real-time PCR assays that detect genes for botulinum neurotoxin A–G subtypes
Source: Front Microbiol. 2024 May 30;15:1382056. doi: 10.3389/fmicb.2024.1382056 (PMC11169944; doi:10.3389/fmicb.2024.1382056)
Supplement: Supplementary file 2 [file Table_9.DOCX]

**Table S2.** Non-neurotoxigenic clostridial species used in specificity testing.

| **Strain** | **Species** | **Strain** | **Species** |
| --- | --- | --- | --- |
| ATCC 12915 | *C. perfringens A* | ATCC 19404 | *C. sporogenes* |
| ATCC 12917 | *C. perfringens A* | ATCC 3584 | *C. sporogenes* |
| ATCC 12918 | *C. perfringens A* | ATCC 10779 | *C. tetani* |
| ATCC 12919 | *C. perfringens A* | ATCC 19406 | *C. tetani* |
| ATCC 12920 | *C. perfringens A* | ATCC 9650 | *C. haemolyticum* |
| ATCC 3629 | *C. perfringens D* | ATCC 25774 | *C. subterminale* |
| ATCC 3630 | *C. perfringens D* | ATCC 25772 | *C. spp (hastiforme)* |
| ATCC 3631 | *C. perfringens D* | ATCC 638 | *C. bifermetnans* |
| ATCC 27324 | *C. perfringens E* | ATCC 17861 | *C. spp.* |
| ATCC 27555 | *C. absonum* | ATCC 25582 | *C. ramosum* |
| ATCC 27638 | *C. baratii* | ATCC 14573 | *C. tertium* |
| ATCC 19401 | *C. histolyticum* | ATCC 3624 | *C. perfringens A* |
| ATCC 27606 | *C. novyi B* | ATCC 3626 | *C. perfringens B* |
| ATCC 19402 | *C. novyi A* | ATCC 13124 | *C. perfringens A* |
| ATCC 12464 | *C. septicum* | ATCC 19402 | *C. novyi* |
| ATCC 9714 | *C. sordelli* |  |  |
