# Supplementary material for: Real-time PCR assays that detect genes for botulinum neurotoxin A–G subtypes
Source: Front Microbiol. 2024 May 30;15:1382056. doi: 10.3389/fmicb.2024.1382056 (PMC11169944; doi:10.3389/fmicb.2024.1382056)
Supplement: Supplementary file 3 [file Table_10.DOCX]

**Table S3.** Bacterial DNA preparations used in specificity testing**.** A panel of DNA preparations representing 110 prokaryotic species was developed consisting of 11 pools with 10 species per pool.

| **Pool** | **Species** |
| --- | --- |
| 1 | *Acidimicrobium ferrooxidans* |
| 1 | *Actinomyces naeslundii* |
| 1 | *Corynebacterium diphtheriae* |
| 1 | *Dietzia maris* |
| 1 | *Mycobacterium smegmatis* |
| 1 | *Frankia sp. AvcI1* |
| 1 | *Blastococcus aggregatus* |
| 1 | *Sporichthya polymorpha* |
| 1 | *Brevibacterium casei* |
| 1 | *Cellulomonas biazotea* |
| 2 | *Brachybacterium faecium* |
| 2 | *Dermatophilus congolensis* |
| 2 | *Intrasporangium calvum* |
| 2 | *Micrococcus luteus* |
| 2 | *Propionibacterium acnes* |
| 2 | *Streptomyces cattleya* |
| 2 | *Thermobifida alba* |
| 2 | *Bifidobacterium infantis* |
| 2 | *Adlercreutzia equolifaciens* |
| 2 | *Conexibacter woesei* |
| 3 | *Aquifex pyrophilus* |
| 3 | *Pedobacter hiparinus* |
| 3 | *Chlorobium ferrooxidans* |
| 3 | *Victivallis vadensis* |
| 3 | *Prosthecobacter debontii* |
| 3 | *Chloroflexus aurantiacus* |
| 3 | *Herpetosiphon aurantiacus* |
| 3 | *Sphaerobacter thermophilus* |
| 3 | *Thermomicrobium roseum* |
| 3 | *Chrysiogenes arsenatis* |
| **Pool** | **Species** |
| 4 | *Tolypothrix tenuis* |
| 4 | *Calditerrivibrio nitroreducens* |
| 4 | *Deinococcus radiodurans* |
| 4 | *Meiothermus cerbereus* |
| 4 | *Dictyoglomus turgidum* |
| 4 | *Acidobacterium capsulatum* |
| 4 | *Pasteuria ramosa* |
| 4 | *Bacillus subtilis* |
| 4 | *Listeria monocytogenes* |
| 4 | *Planococcus citreus* |
| 5 | *Marinococcus albus* |
| 5 | *Staphylococcus aureus* |
| 5 | *Aerococcus viridans* |
| 5 | *Enterococcus asini* |
| 5 | *Lactobacillus acidophilus* |
| 5 | *Leuconostoc mesenteroides* |
| 5 | *Streptococcus pyogenes* |
| 5 | *Clostridium perfringens* |
| 5 | *Eubacterium limosum* |
| 5 | *Helcococcus kunzii* |
| 6 | *Halanaerobium saccharolyticum* |
| 6 | *Thermoanaerobacter subterraneus* |
| 6 | *Anaeroplasma sp. DSMZ 3268* |
| 6 | *Holdemania filiformis* |
| 6 | *Spiroplasma cantharicola* |
| 6 | *Mycoplasma orale* |
| 6 | *Fusobacterium simiae* |
| 6 | *Gemmatimonas aurantiaca* |
| 6 | *Leptospirillum ferriphilum* |
| 6 | *Pirellula marina* |
| **Pool** | **Species** |
| 7 | *Asticcacaulis biprosthecium* |
| 7 | *Agromonas oligotrophica* |
| 7 | *Brucella abortus* |
| 7 | *Methylopila capsulata* |
| 7 | *Hoeflea marina* |
| 7 | *Rhizobium arachis* |
| 7 | *Xanthobacter agilis* |
| 7 | *Hyphomonas neptunium* |
| 7 | *Paracoccus versutus* |
| 7 | *Acidiphilium acidophilum* |
| 8 | *Rickettsia prowazekii* |
| 8 | *Erythrobacter longus* |
| 8 | *Zymomonas mobilis* |
| 8 | *Bordetella pertussis* |
| 8 | *Ralstonia solanacearum* |
| 8 | *Hydrogenophilus hirschii* |
| 8 | *Methylobacillus flagellatus* |
| 8 | *Nitrosomonas cryotolerans* |
| 8 | *Azoarcus communis* |
| 8 | *Desulfatibacillum alkenivorans* |
| 9 | *Desulfohalobium utahense* |
| 9 | *Hippea maritima* |
| 9 | *Desulfuromonas thiophila* |
| 9 | *Enhygromyxa salina* |
| 9 | *Desulfobacca acetoxidans* |
| 9 | *Arcobacter halophilus* |
| 9 | *Caminibacter mediatlanticus* |
| 9 | *Acidithiobacillus albertensis* |
| 9 | *Aeromonas hydrophila* |
| 9 | *Alteromonas macleodii* |
| **Pool** | **Species** |
| 10 | *Nitrosococcus oceani* |
| 10 | *Arsenophonus nasoniae* |
| 10 | *Escherichia coli* |
| 10 | *Aquicella siphonis* |
| 10 | *Legionella pneumophila* |
| 10 | *Alcanivorax jadensis* |
| 10 | *Moraxella lacunata* |
| 10 | *Pseudomonas putida* |
| 10 | *Methylophaga alcalica* |
| 10 | *Vibrio fischeri* |
| 11 | *Aquimonas voraii* |
| 11 | *Leptonema illini* |
| 11 | *Fervidobacterium islandicum* |
| 11 | *Bordetella bronchiseptica* |
| 11 | *Salmonella typhi* |
| 11 | *Pseudomonas aeruginosa* |
| 11 | *Treponema denticola* |
| 11 | *Bacillus thuringiensis* |
| 11 | *Streptococcus pneumoniae* |
| 11 | *Lactobacillus plantarum* |
|  |  |
|  |  |
|  |  |
|  |  |
|  |  |
|  |  |
|  |  |
|  |  |
|  |  |
