# Supplementary material for: Real-time PCR assays that detect genes for botulinum neurotoxin A–G subtypes
Source: Front Microbiol. 2024 May 30;15:1382056. doi: 10.3389/fmicb.2024.1382056 (PMC11169944; doi:10.3389/fmicb.2024.1382056)
Supplement: Supplementary file 4 [file Table_11.DOCX]

**Table S4.** Fungal, yeast and protozoan DNA preparations used in specificity testing. Three pools of DNA preparations representing four fungal species, four species of yeast, and four protozoan species were used.

| **Pool** | **Fungi and yeast** |
| --- | --- |
| 1 | *Aspergillus niger* |
| 1 | *Candida albicans* |
| 1 | *Cryptococcus laurentii* |
| 1 | *Cryptococcus neoformans* |
| 2 | *Penicillium marneffei* |
| 2 | *Pichia stipitis* |
| 2 | *Saccharomyces cerevisiae* |
| 2 | *Schizosaccharomyces pombe* |
| **Pool** | **Protozoa** |
| 3 | *Acanthamoeba castellanii* |
| 3 | *Giardia intestinalis* |
| 3 | *Toxoplasma gondii* |
| 3 | *Cryptosporidium parvum* |
