# Supplementary material for: Real-time PCR assays that detect genes for botulinum neurotoxin A–G subtypes
Source: Front Microbiol. 2024 May 30;15:1382056. doi: 10.3389/fmicb.2024.1382056 (PMC11169944; doi:10.3389/fmicb.2024.1382056)
Supplement: Supplementary file 5 [file Table_12.DOCX]

**Table S5.** DNA preparations from animals and plants used in specificity testing. Six pools of DNA preparations representing 21 animals and four plants were used.

| **Pool** | **Higher eukaryotes** | **Pool** | **Higher eukaryotes** |
| --- | --- | --- | --- |
| 1 | Catfish | 4 | Dog |
| 1 | Chicken | 4 | Goat |
| 1 | Turkey | 4 | Sheep |
| 1 | Goose | 4 | Bovine |
| 2 | Mouse | 5 | Porcine |
| 2 | Gerbil | 5 | Donkey |
| 2 | Hamster | 5 | Horse |
| 2 | Guinea pig | 5 | Human male |
| 3 | Rat | 6 | Rhesus Monkey |
| 3 | Rabbit | 6 | Corn |
| 3 | Ferret | 6 | Wheat |
| 3 | Cat | 6 | Rice |
|  |  | 7 | Soybeans |
