# Supplementary material for: Real-time PCR assays that detect genes for botulinum neurotoxin A–G subtypes
Source: Front Microbiol. 2024 May 30;15:1382056. doi: 10.3389/fmicb.2024.1382056 (PMC11169944; doi:10.3389/fmicb.2024.1382056)
Supplement: Supplementary file 6 [file Table_13.DOCX]

**Table S6.** Experimental data from testing primers and probes with the target DNA. Sensitivity testing was performed with 1 pg of DNA per assay and specificity testing was done at 100 pg of DNA per assay. Selected results from testing at 100 pg of DNA are listed for comparative purposes and to illustrate assay differences at different DNA quantities. Results are shown in Ct values; values of less than 40 are considered positive. Positive results with the VIC dye probe of the Cb(stop) assay illustrate this assay’s ability to detect the *bont* gene encoding active BoNT/B1, /B2, /B4, and /B5, and results with the FAM dye probe allow for differentiation between *bont*/(B) and *bont*/B genes. U = undetected.

| **A Strain** | **Subtype** | **Amount of DNA tested** | **CbA**  **2.323** | **CbA**  **4.16566** | **CbA**  **2.341** | **16S rRNA** |
| --- | --- | --- | --- | --- | --- | --- |
| Schantz | A1 | 1 pg | 36.4 | 36.2 | 35.6 | 33.6 |
| ATCC 3502 | A1 | 1 pg | 36.7 | 35.5 | 35.3 | 34.5 |
| ATCC 17862 | A1 | 1 pg | 35.1 | 34.8 | 33.4 | 36.1 |
| ATCC 19397 | A1 | 1 pg | 35.7 | 36.8 | 34.8 | 34.8 |
| ATCC 25763 | A1 | 1 pg | 37.4 | 36.7 | 36.2 | 34.2 |
| CDC 1757 | A1 | 1 pg | 37.2 | 37.1 | 36.2 | 33.6 |
| Hall | A1 | 1 pg | 36.3 | 35.9 | 35.4 | 35.2 |
| Hall 5675 | A1 | 1 pg | 36.8 | 36.6 | 36.4 | 34.6 |
| Prevot Ppois | A1 | 1 pg | 35.9 | 35.9 | 35.3 | 33.4 |
| ATCC 449 | A1 | 1 pg | 33.6 | 33.7 | 32.2 | 31.2 |
| Hall 11481 | A1 | 1 pg | 36.2 | 37.9 | 35.7 | 33.4 |
| CDC 1744 | A1(B) | 1 pg | 37.0 | 32.5 | 36.0 | 33.5 |
| Hall 183 | A1(B) | 1 pg | 34.2 | 35.2 | 33.1 | 32.8 |
| Hall 3685a | A1(B) | 1 pg | 36.5 | 36.3 | 35.0 | 36.5 |
| CDC 2357 | A1(B) | 1 pg | 36.6 | 34.6 | 35.1 | 34.0 |
| FRI honey | A2 | 1 pg | 37.1 | 37.0 | 36.2 | 36.4 |
| Kyoto-F | A2 | 1 pg | 36.9 | 36.7 | 35.3 | 34.5 |
| CDC 1436 | A2/B5 | 1 pg | 36.9 | 37.6 | 35.6 | 34.0 |
| Loch Maree | A3 | 1 pg | 35.7 | 36.4 | 34.5 | 33.9 |
| CDC 657 | B5/A4 | 1 pg | 35.3 | 35.7 | 33.3 | 36.0 |
| Hall 3676 | A1(B) | 1 pg | 33.5 | 33.1 | 32.3 | 34.5 |
| Hall 3676 | A1(B) | 100 pg | 27.9 | 28.0 | 27.3 | 24.4 |

| **B Strain** | **Subtype** | **Amount of DNA tested** | **CbB**  **2.11** | **CbB**  **2.295** | **16S rRNA** |
| --- | --- | --- | --- | --- | --- |
|  |  |  |  |  |  |
| beans | B1 | 1 pg | 37.5 | 39.0 | 33.7 |
| okra | B1 | 1 pg | 34.4 | 34.6 | 36.0 |
| CDC 1656 | B1 | 1 pg | 36.2 | 36.3 | 34.5 |
| CDC 1758 | B1 | 1 pg | 36.6 | 36.4 | 33.6 |
| ATCC 17783 | B1 | 1 pg | 34.6 | 34.3 | 33.2 |
| L. Ds. Smith | B1 | 1 pg | 35.6 | 36.1 | 37.7 |
| Hall 6517 | B1 | 1 pg | 34.1 | 34.1 | 36.1 |
| CDC 6242 | B1 | 1 pg | 33.6 | 33.8 | 36.8 |
| ATCC 17843 | B2 | 1 pg | 35.8 | 35.8 | 37.0 |
| 213B | B2 | 1 pg | 35.0 | 34.9 | 34.6 |
| CDC 1828 | B2 | 1 pg | 36.1 | 36.7 | 34.4 |
| Prevot 892 | B2 | 1 pg | 33.4 | 33.2 | 34.8 |
| Prevot 59 | B2 | 1 pg | 36.0 | 35.5 | 34.7 |
| Prevot 25NCASE | B2 | 1 pg | 34.8 | 34.7 | 34.8 |
| Prevot 1740 | B2 | 1 pg | 34.2 | 34.1 | 33.8 |
| Prevot B | B2 | 1 pg | 33.2 | 32.6 | 35.3 |
| Prevot 314A | B2 | 1 pg | 34.4 | 34.1 | 35.6 |
| Smith L-590 | B2 | 1 pg | 35.7 | 35.3 | 34.1 |
| ATCC 8083 | B2 | 1 pg | 34.9 | 34.8 | 33.8 |
| CDC 6291 | B2 | 1 pg | 36.4 | 36.1 | 35.6 |
| Prevot 2345 | B2 | 1 pg | 34.7 | 34.5 | 34.9 |
| An436 | B5F2 | 1 pg | 37.3 | 37.7 | 34.8 |
| CDC3281 | B5F2 | 1 pg | 36.5 | 36.3 | 33.2 |
| Eklund 17B | B4 | 1 pg | 34.7 | 34.9 | 37.3 |
| ATCC 17844 | B4 | 1 pg | 32.2 | 32.6 | 37.9 |
| 100688 | B4 | 1 pg | 33.7 | 34.2 | 37.7 |
| CDC 1436 | A2B5 | 1 pg | 36.5 | 36.8 | 38.4 |
| CDC 657 | B5A4 | 1 pg | 36.5 | 36.3 | 36.8 |
| CDC 1744 | A1(B) | 1 pg | 35.7 | 36.6 | 34.9 |
| Hall 183 | A1(B) | 1 pg | 33.2 | 33.2 | 33.2 |
| Hall 3676 | A1(B) | 1 pg | 32.5 | 32.5 | 32.7 |
| Hall 3685a | A1(B) | 1 pg | 35.9 | 35.3 | 36.4 |
| CDC 2357 | A1(B) | 1 pg | 36.2 | 36.1 | 35.0 |
| L. Ds. Smith | B1 | 1 pg | 35.6 | 36.1 | 37.7 |
| L. Ds. Smith | B1 | 100 pg | 26.8 | 26.8 | 24.4 |

| **(B) Strain** | **Subtype** | **Amount of DNA tested** | **Cb(B)stop** | | **16S rRNA** |
| --- | --- | --- | --- | --- | --- |
|  |  |  | **FAM** | **VIC** |  |
| CDC 1744 | A1(B) | 1 pg | 30.8 | U | 29.1 |
| Hall 183 | A1(B) | 1 pg | 33.3 | U | 33.6 |
| Hall 3676 | A1(B) | 1 pg | 31.5 | U | 31.7 |
| Hall 3685a | A1(B) | 1 pg | 34.4 | U | 35.5 |
| CDC 2357 | A1(B) | 1 pg | 32.5 | U | 32.5 |
| beans | B1 | 100 pg | U | 31.6 | 22.8 |
| okra | B1 | 100 pg | U | 28.0 | 23.8 |
| CDC 1656 | B1 | 100 pg | U | 28.0 | 24.6 |
| CDC 1758 | B1 | 100 pg | U | 29.1 | 27.5 |
| ATCC 17783 | B1 | 100 pg | U | 27.8 | 25.3 |
| L. Ds. Smith | B1 | 100 pg | U | 29.3 | 24.4 |
| Hall 6517 | B1 | 100 pg | U | 26.5 | 22.4 |
| CDC 6242 | B1 | 100 pg | U | 27.7 | 23.3 |
| ATCC 17843 | B2 | 100 pg | U | 28.6 | 25.6 |
| 213B | B2 | 100 pg | U | 27.3 | 25.2 |
| CDC 1828 | B2 | 100 pg | U | 30.0 | 26.8 |
| Prevot 892 | B2 | 100 pg | U | 27.1 | 24.5 |
| Prevot 59 | B2 | 100 pg | U | 26.3 | 25.3 |
| Prevot 25NCASE | B2 | 100 pg | U | 28.9 | 23.8 |
| Prevot 1740 | B2 | 100 pg | U | 27.7 | 23.7 |
| Prevot B | B2 | 100 pg | U | 27.4 | 23.5 |
| Prevot 314A | B2 | 100 pg | U | 28.1 | 23.4 |
| Smith L-590 | B2 | 100 pg | U | 26.9 | 23.4 |
| ATCC 8083 | B2 | 100 pg | U | 25.8 | 23.4 |
| CDC 6291 | B2 | 100 pg | U | 30.2 | 24.7 |
| Prevot 2345 | B2 | 100 pg | U | 29.0 | 23.5 |
| An436 | B5F2 | 100 pg | U | 27.4 | 24.1 |
| CDC3281 | B5F2 | 100 pg | U | 27.5 | 24.6 |
| Eklund 17B | B4 | 100 pg | U | 27.7 | 26.2 |
| ATCC 17844 | B4 | 100 pg | U | 25.4 | 26.2 |
| 100688 | B4 | 100 pg | U | 25.8 | 22.8 |
| CDC 1436 | A2B5 | 100 pg | U | 27.1 | 23.8 |
| CDC 657 | B5A4 | 100 pg | U | 26.8 | 23.9 |

| **C Strain** | **Subtype** | **Amount of DNA tested** | **CbC**  **2.11** | **CbC**  **2.968** | **16S rRNA** |
| --- | --- | --- | --- | --- | --- |
|  |  |  |  |  |  |
| Stockholm | C | 1 pg | 37.9 | 36.8 | 34.5 |
| 2048-Mich | C | 1 pg | 34.5 | 33.6 | 32.0 |
| ATCC 17849 | C | 1 pg | 37.0 | 36.6 | 31.7 |
| ATCC 17784 | C | 1 pg | 32.9 | 32.8 | 30.2 |
| 003-9 | CD | 1 pg | 34.7 | 34.8 | 30.5 |
| 468 | C | 1 pg | 37.1 | 36.4 | 32.6 |
| Copenhagen 41/59-60* | C | 1 pg | 38.2 | 37.0 | 33.3 |
| 6816 | CD | 1 pg | 34.8 | 34.6 | 31.2 |
| Prevot 571Y | C | 1 pg | 32.4 | 32.3 | 29.1 |
| Prevot 2266 | C | 1 pg | 32.5 | 32.6 | 30.0 |
| Copenhagen 41/59-60* | C | 1 pg | 33.1 | 32.7 | 30.8 |
| Copenhagen 41/59-60 | C | 100 pg | 27.5 | 28.0 | 25.2 |

*these samples represent distinct subclones of the same strain

| **D Strain** | **Subtype** | **Amount of DNA tested** | **CbD**  **2.0** | **CbD**  **2.276** | **16S rRNA** |
| --- | --- | --- | --- | --- | --- |
|  |  |  |  |  |  |
| Schantz | D | 1 pg | 37.6 | 38.8 | 31.4 |
| ATCC 11873 | D | 1 pg | 35.0 | 36.0 | 31.9 |
| 1873 | D | 1 pg | 36.2 | 37.5 | 31.4 |
| 1873 | D | 100 pg | 29.1 | 29.2 | 24.1 |

| **E Strain** | **Subtype** | **Amount of DNA tested** | **CbE**  **2.0** | **CbE**  **2.693** | **16S rRNA** |
| --- | --- | --- | --- | --- | --- |
|  |  |  |  |  |  |
| Beluga | E1 | 1 pg | 36.0 | U/39.1/37.2 | 35.1 |
| Prevot Ped1 | E1 | 1 pg | 35.0 | 37.4 | 33.5 |
| ATCC 9564 | E1 | 1 pg | 35.2 | 38.1 | 35.4 |
| CDC 5247 | E2 | 1 pg | 34.4 | 36.1 | 32.1 |
| CDC 5906 | E2 | 1 pg | 37.1 | 39.5/U/35.8 | 34.1 |
| CDC 5258 | E3 | 1 pg | 35.3 | 37.6 | 31.6 |
| Prevot R81-3A | E3 | 1 pg | 35.2 | 36.7 | 33.1 |
| BL5262 | E4 | 1 pg | 36.0 | U/39.8/U | 33.8 |
| ATCC 17852 | E1 | 1 pg | 35.8 | U | 34.0 |
| ATCC 17852 | E1 | 100 pg | 27.0 | 29.9 | 24.3 |

| **F Strain** | **Subtype** | **Amount of DNA tested** | **CbF**  **2.0** | **CbF**  **2.161** | **CbF**  **2.1084** | **16S rRNA** |
| --- | --- | --- | --- | --- | --- | --- |
| Langeland | F1 | 1 pg | 35.4 | U | U | 33.8 |
| 6/14 | F1 | 1 pg | 35.3 | U | U | 35.1 |
| Walls 8-G | F1 | 1 pg | 34.6 | U | U | 33.5 |
| Eklund202F | F6 | 1 pg | 35.6 | U | U | 35.6 |
| CDC 3281 | B5F2 | 1 pg | 34.1 | U | 35.2 | 33.2 |
| CDC 2821 | F1 | 1 pg | 35.1 | U | U | 32.8 |
| CDC 2821 | F1 | 100 pg | 27.8 | U | U | 22.6 |
| An436 | B5F2 | 1 pg | 31.0 | U | 31.6 | 28.9 |
| An436 | B5F2 | 100 pg | 27.3 | U | 28.4 | 24.1 |
| Sullivan | F7 | 1 pg | U | 37.0 | U | 32.1 |
| Sullivan | F7 | 100 pg | U | 29.9 | U | 23.3 |

| **G Strain** | **Subtype** | **Amount of DNA tested** | **CbG**  **2.0** | **CbG**  **2.83** | **16S rRNA** |
| --- | --- | --- | --- | --- | --- |
|  |  |  |  |  |  |
| CDC 2738 (SN 143/77) | G | 1 pg | U | 36.1 | 34.0 |
| 1354 | G | 1 pg | U | 36.9 | 33.9 |
| CDC 2739 (GM 140/77) | G | 1 pg | U | 38.3 | 35.6 |
| CDC 2740 (GM 73/78) | G | 1 pg | U | 38.2 | 35.2 |
| CDC 2742 (GM 56/78) | G | 1 pg | U | 36.8 | 33.1 |
| CDC 2741 (GM 77/78) | G | 1 pg | U | 37.9 | 33.5 |
| CDC 2741 (GM 77/78) | G | 100 pg | 28.3 | 27.4 | 26.5 |
