# Supplementary material for: Real-time PCR assays that detect genes for botulinum neurotoxin A–G subtypes
Source: Front Microbiol. 2024 May 30;15:1382056. doi: 10.3389/fmicb.2024.1382056 (PMC11169944; doi:10.3389/fmicb.2024.1382056)
Supplement: Supplementary file 7 [file Table_14.DOCX]

**Table S7.** Summaries of *in silico* analyses of ISO/TC 17919:2013 by subtype. Predicted positive results are those having exact matches with both primers and the probe; negative results show one or more mismatches in either the primers or probe. Numbers in parentheses () are instances where the mismatches were so great that the sequences were absent from the analysis results.

|  |  | **CBOT A** | | **Fach Type A** | | **CBMLA** | |
| --- | --- | --- | --- | --- | --- | --- | --- |
| **A** |  | predicted positive | predicted negative | predicted positive | predicted negative | predicted positive | predicted negative |
| Subtype | # analyzed | (exact match) | (mismatches) | (exact match) | (mismatches) | (exact match) | (mismatches) |
| A1 | 125 | 125 | 0 | 125 | 0 | 125 | 0 |
| A1(B) | 61 | 61 | 0 | 61 | 0 | 61 | 0 |
| A2 | 148 | 147 | 1 | 147 | 1 | 148 | 0 |
| A3 | 12 | 12 | 0 | 0 | 12 | 0 | 12 |
| A4 | 3 | 3 | 0 | 0 | 3 | 0 | 3 |
| A5 | 6 | 6 | 0 | 6 | 0 | 6 | 0 |
| A6 | 2 | 2 | 0 | 2 | 0 | 2 | 0 |
| A7 | 1 | 1 | 0 | 0 | 1 | 1 | 0 |
| A8 | 2 | 2 | 0 | 2 | 0 | 2 | 0 |
|  |  |  |  |  |  |  |  |
|  |  | **IA** | |  |  |  |  |
|  | | predicted positive | predicted negative |  |  |  |  |
| Subtype | # analyzed | (exact match) | (mismatches) |  |  |  |  |
| A1 | 125 | 112 | 13 |  |  |  |  |
| A1(B) | 61 | 62 | 0 |  |  |  |  |
| A2 | 148 | 147 | 1 |  |  |  |  |
| A3 | 12 | 12 | 0 |  |  |  |  |
| A4 | (3) | 0 | (3) |  |  |  |  |
| A5 | 6 | 0 | 6 |  |  |  |  |
| A6 | 2 | 0 | 2 |  |  |  |  |
| A7 | 1 | 0 | 1 |  |  |  |  |
| A8 | 2 | 2 | 0 |  |  |  |  |
|  |  |  |  |  |  |  |  |

|  |  |  |  |  |  |  |  |
| --- | --- | --- | --- | --- | --- | --- | --- |
|  |  | **CBOT B** | | **Fach Type B** | | **CBMLB** | |
| **B** |  | predicted positive | predicted negative | predicted positive | predicted negative | predicted positive | predicted negative |
| Subtype | # analyzed | (exact match) | (mismatches) | (exact match) | (mismatches) | (exact match) | (mismatches) |
| A1(B) | 62 | 62 | 0 | 62 | 0 | 62 | 0 |
| B1 | 9 | 9 | 0 | 9 | 0 | 9 | 0 |
| B2 | 91 | 91 | 0 | 91 | 0 | 91 | 0 |
| B3 | 32 | 32 | 0 | 32 | 0 | 32 | 0 |
| B4 | 73 | 0 | 73 | 58 | 15 | 1 | 72 |
| B5 | 19 | 19 | 0 | 19 | 0 | 19 | 0 |
| B6 | 5 | 5 | 0 | 4 | 1 | 0 | 5 |
| B7 | 11 | 11 | 0 | 0 | 11 | 11 | 0 |
| B8 | 3 | 0 | 3 | 0 | 3 | 0 | 3 |
|  |  |  |  |  |  |  |  |
|  |  |  |  |  |  |  |  |
|  |  | **CBOT E** | | **Fach Type E** | | **CBMLE** | |
| **E** |  | predicted positive | predicted negative | predicted positive | predicted negative | predicted positive | predicted negative |
| Subtype | # analyzed | (exact match) | (mismatches) | (exact match) | (mismatches) | (exact match) | (mismatches) |
| E1 | 79 | 79 | 0 | 79 | 0 | 78 | 1 |
| E2 | 5 | 5 | 0 | 5 | 0 | 5 | 0 |
| E3 | 106 | 106 | 0 | 106 | 0 | 105 | 1 |
| E4 | 6 | 6 | 0 | 6 | 0 | 0 | 6 |
| E5 | 15 | 15 | 0 | 15 | 0 | 15 | 0 |
| E6 | 8 | 8 | 0 | 8 | 0 | 8 | 0 |
| E7 | 2 | 2 | 0 | 2 | 0 | 0 | 2 |
| E8 | 1 | 0 | 1 | 0 | 1 | 0 | 1 |
| E9 | 1 | 0 | 1 | 0 | 1 | 0 | 1 |
| E10 | 45 | 45 | 0 | 45 | 0 | 0 | 45 |
| E11 | 9 | 0 | 9 | 0 | 9 | 0 | 9 |
| E12 | 1 | 0 | 1 | 0 | 1 | 0 | 1 |
|  |  |  |  |  |  |  |  |

|  |  |  |  |  |  |  |  |
| --- | --- | --- | --- | --- | --- | --- | --- |
|  |  | **CBOT F** | | **Type F** | | **CBMLF** | |
| **F** |  | predicted positive | predicted negative | predicted positive | predicted negative | predicted positive | predicted negative |
| Subtype | # analyzed | (exact match) | (mismatches) | (exact match) | (mismatches) | (exact match) | (mismatches) |
| F1 | 16 | 16 | 0 | 16 | 0 | 16 | 0 |
| F2 | 14 | 0 | 14 | 0 | 14 | 0 | 14 |
| F3 | 3 | 0 | 3 | 3 | 0 | 0 | 3 |
| F4 | 19 | 19 | 0 | 19 | 0 | 0 | 19 |
| F5 | 4 | 0 | 4 | 4 | 0 | 0 | 4 |
| F6 | 18 | 18 | 0 | 17 | 1 | 0 | 18 |
| F7 | 12 | 0 | 12 | 0 | 12 | 0 | 12 |
| F8 | 1 | 0 | 1 | 1 | 0 | 0 | 1 |
| F9 | 1 | 0 | 1 | 0 | 1 | 0 | 1 |
